# The protein kinase IKK epsilon shapes the melanoma tumor microenvironment by modulating angiogenesis and the T-cell immunity

**DOI:** 10.1186/s10020-026-01651-w

**Published:** 2026-09-30

**Authors:** Michelle Haß, Eleonora Mungo, Denis Benning, Andreas Weigert, Blerina Aliraj, Josefine Jakob, Björn Häupl, Thomas Oellerich, Nicole Ziegler, Aimo Kannt, Gerd Geisslinger, Ellen Niederberger

**Affiliations:** 1 Goethe-University Frankfurt, pharmazentrum frankfurt/ZAFES, Faculty of Medicine, Institute of Clinical Pharmacology, Theodor Stern Kai 7, Frankfurt Am Main, 60590 Germany; 2 Goethe-University Frankfurt, Faculty of Medicine, Institute of Biochemistry I, Theodor Stern Kai 7, Frankfurt am Main, 60590 Germany; 3 Department for Immunity of Inflammation, Mannheim Institute for Innate Immunoscience (MI3), Medical Faculty Mannheim, Heidelberg University, Mannheim, Germany; 4 Goethe University Frankfurt, University Hospital, Department of Medicine, Hematology and Oncology, Frankfurt Am Main, Germany; 5 Frankfurt Cancer Institute (FCI), Frankfurt Am Main, Germany; 6 German Cancer Consortium (DKTK), Partner Site Frankfurt/Mainz, a Partnership Between DKFZ and UCT Frankfurt-Marburg, Frankfurt Am Main, Germany; 7 German Cancer Research Center (DKFZ), Heidelberg, Germany; 8 University Cancer Center (UCT), Frankfurt Am Main, Germany; 9 Fraunhofer Institute for Translational Medicine and Pharmacology ITMP, Sandhöfer Allee 1, Frankfurt Am Main, 60528 Germany

**Keywords:** Melanoma, IKKε, Tumor growth, T-cells, angiogenesis

## Abstract

**Background:**

Inhibitor of nuclear factor kappa-B kinase epsilon (IKKε) contributes to tumorigenesis and metastasis in various cancers. In melanoma, it is overexpressed and constitutively active. Inhibition of IKKε by either knockdown or pharmaceutical compounds reduce tumor progression by suppressing key molecular pathways within melanoma tumor cells. However, within the tumor microenvironment (TME), the role of this protein remains to be fully elucidated.

**Methods:**

Multiplex immunohistochemistry was performed on human primary melanomas to assess IKKε expression in tumor and immune cell compartments. In the mouse model, B16BL6 melanoma cells were injected subcutaneously into the flanks of wildtype and IKKε-deficient mice, and tumor growth was monitored over time. Flow-cytometric analyses characterized intratumoral immune cell populations in both genotypes, followed by in vivo CD8⁺ T-cell depletion to determine their contribution to tumor control. To elucidate molecular mechanisms underlying the differential tumor growth, we conducted targeted and untargeted proteomic analyses, which indicated regulation of angiogenic pathways which were further examined by assessing specific protein changes in tumor tissue and performing in vitro angiogenesis assays.

**Results:**

The reduced tumor growth observed in IKKε knock-out (KO) mice was concomitant with elevated levels of cytotoxic CD8⁺ and γδ-T-cells, and a diminished presence of neutrophils. Furthermore, our data revealed that isolated CD8⁺ T-cells from IKKε KO mice exhibited a higher activity in comparison to their wildtype counterparts. In addition, the depletion of CD8⁺ cells reversed the antitumor efficacy of IKKε depletion.

Proteomic analysis of tumor lysates revealed a reduction of several chemokines in IKKε KO mice compared to wildtype controls, that might contribute to the reduced tumor growth and suggested that an inhibition of angiogenesis may support the antitumor activity in IKKε KO mice. This hypothesis could be confirmed by further protein analyses and angiogenesis assays.

**Conclusions:**

Our data imply that IKKε expression in melanoma is not only important in the tumor cells but also in the tumor microenvironment by regulation of the immune response and angiogenesis. Consequently, inhibition of IKKε may offer a novel therapeutic approach for melanoma, complementing existing therapies that target pathways within tumor cells and antitumor immune responses in the tumor microenvironment.

**Graphical Abstract:**

**Figure:**
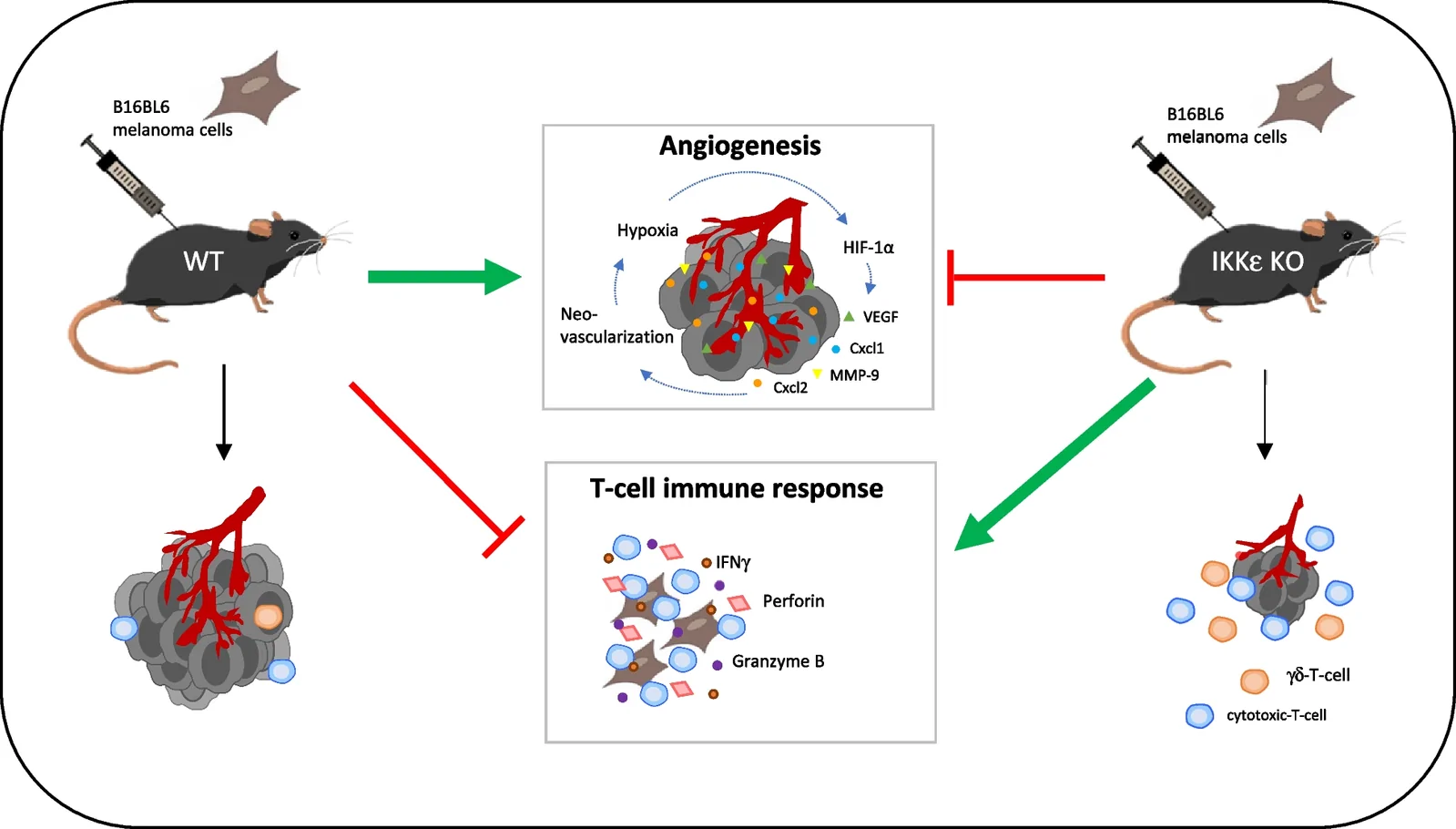

**Supplementary Information:**

The online version contains supplementary material available at https://doi.org/10.1186/s10020-026-01651-w.

## Introduction

Malignant melanoma is characterized by its highly metastatic potential and is the most aggressive form of skin cancer with an increasing global incidence. Its therapy is predominantly based on surgical excision of the tumor while pharmacological treatment is rendered challenging. Drugs used for melanoma therapy comprise BRAF/MEK inhibitors, as well as immunotherapeutic options that target immune checkpoints, such as ipilimumab and nivolumab (Alsaab et al. 2017; Luke et al. 2017). However, several melanomas quickly develop resistance against the available drugs or do not react to available immunotherapies. Consequently, the five-year survival rates of patients diagnosed with advanced melanoma are very poor (Long et al. 2017; Matthews et al. 2017). Therefore, there is a substantial need to develop new treatment strategies based on alternative molecular mechanisms of melanoma initiation and progression in the tumor itself as well as in immunoregulatory pathways in the tumor microenvironment.

The IKK-related kinases IKKε and TANK-binding kinase 1 (TBK1) are involved in the activation of the transcription factor NF-κB (Adli and Baldwin 2006; Mattioli et al. 2006; Moser et al. 2011; Peters and Maniatis 2001; Tojima et al. 2000) and have already been associated with different tumors, including melanoma (Baud and Karin 2009; Dhawan et al. 2002; McNulty et al. 2004) (reviewed in (Clement et al. 2008; Shen and Hahn 2011)). We could show that IKKε is overexpressed in murine and human malignant melanoma cells in comparison to melanocytes, as well as in metastases of melanoma patients (Möller et al. 2020; Moser et al. 2016). As demonstrated in the Human Cancer Atlas, there is a correlation between elevated IKKε expression and a reduced survival rate among melanoma patients, in comparison to individuals exhibiting low IKKε expression (https://www.proteinatlas.org/humanproteome/cancer). Our preclinical findings further indicate that the inhibition of IKKε in melanoma tumor cells is associated with a reduction in cell proliferation and results in altered regulation of NF-κB, Akt1 and mitogen-activated protein kinase (MAPK) pathways (Moser et al. 2016). In a mouse xenograft model, inhibition of IKKε by the drug amlexanox significantly suppressed melanoma growth by inhibiting MAPK and autophagy processing (Moller et al. 2020). Other studies hypothesized that inhibitors of IKKε/TBK1 might be efficacious in the treatment of therapy-resistant melanoma (Eskiocak et al. 2017). Furthermore, our data indicated that IKKε is expressed not only in melanoma cells in the tumor but also in a large proportion of immune cells, in particular T-cells (Moller et al., 2020). This finding is consistent with data of other studies, which demonstrated abundant expression of IKKε in T-cells (Peters et al. 2000) and an increase in IKKε activity following T-cell activation which is associated with negative feedback inhibition of T-cell activity by IKKε-mediated phosphorylation of nuclear factor of activated T-cells (NFAT). IKKε-depleted mice exhibited a higher survival rate and reduced tumor development than wildtype mice following intravenous injection of melanoma cells probably due to an increase of T-cell-mediated anti-tumor immunity (Zhang et al. 2016). These data suggest a potential involvement of IKKε in several regulatory processes, both within melanoma cells and cells present within the tumor microenvironment. However, the precise mechanisms underlying the inhibition of melanoma growth remain to be fully elucidated. The present study thus concentrated on the yet unidentified mechanisms of IKKε in the tumor microenvironment which contribute to the growth and progression of melanoma.

## Materials and methods

### Animals

Homozygous IKKε^−/−^-mice with a C57BL/6 background were purchased from The Jackson Laboratories, USA (B6.Cg-Ikbke^tm1Tman^/J). In these mice, the exons 4-6 of the IKKε gene were replaced by a PGK-neo cassette resulting in an inactive protein. IKKε^−/−^ mice are viable, fertile and show no obvious abnormalities (Hemmi et al. 2004). Respective age-matched C57BL/6 wildtype mice were purchased from Charles River, Germany. Control genotyping was performed using the following primers as recommended by The Jackson Laboratories:

**Table:** 

*Primer 5' *	*Sequence 5' 3'*	*Primer Type*
oIMR6916	CTT GGG TGG AGA GGC TAT TC	Mutant Forward
oIMR6917	AGG TGA GAT GAC AGG AGA TC	Mutant Reverse
oIMR7048	GGC CCA CCG AAG GGG ATG AAG G	Wildtype Forward
oIMR7049	CTG CCC GCA AGC TGG ACG ATG AT	Wildtype Reverse

Animals had free access to food and water and were maintained in climate- and light-controlled rooms (24 ± 0.5 °C, 12/12 dark/light cycle). In all experiments the European ethic guidelines for investigations in conscious animals were obeyed and the procedures were approved by the local Ethics Committee for Animal Research (FU/2045). All efforts were made to minimize animal suffering and to reduce the number of animals used.

### Mouse melanoma model

C57BL/6 mice as well as IKKε KO mice at the age of 12 to 16 weeks were inoculated subcutaneously with tumor cells (1 × 10^5^ B16BL6 melanoma cells) in 50 µl Hanks’ Balanced Salt Solution (HBSS +/+) into the left and right flank under brief isoflurane anesthesia. Melanoma growth is first observable as black nodule about 5-7 days after inoculation. The tumor size was determined three times a week using a caliper until the end of the observation period at day 21 or when the tumor size reached 1 cm^3^. Tumor volume was calculated using the V = length x width^2^ x π/6. At the end of the experiment, animals were deeply anesthetized with CO_2_ and killed by cardiac puncture.

### Concurrent T-cell immune depletion protocol

For in vivo depletion of CD8⁺ T-cells, a depleting antibody (InVivoPlus anti-mouse CD8α, bioxcell, West Lebanon, NH, *#* BP0117) or an isotype control IgG (InVivoPlus rat IgG2b isotype control, bioxcell, West Lebanon, NH, # BP0090) were injected intraperitoneally once a week (day 0, 7, 14) at a dose of 250 µg/mouse, starting simultaneously with the inoculation of the tumor cells (day 0) (Fig. 5A). The antibodies were diluted in the buffer recommended by the manufacturer (In VivoPure pH 6.0 T Dilution Buffer, bioxcell, West Lebanon, NH, #IPT060) immediately prior to injection. The final injection volume was 100 to 150 µL. Successful depletion was subsequently verified by flow cytometry of spleen and tumor tissue (antibodies see Suppl. Table 1) which were harvested from the mice at the end of the experiments.

### Flow cytometry

At the end of the experiment, tumors were dissected and weighed. Single-cell suspensions were prepared with the Tumor Dissociation Kit (#130–096–730) and gentleMACS Dissociator (both Miltenyi Biotec, Bergisch Gladbach, Germany) according to the manufacturer’s recommendations. Prior staining, samples were filtered through 70 µm cell strainers (BD Biosciences, Heidelberg, Germany). To prepare single-cell suspensions of spleens, these were disintegrated using a 100 µm cell strainer (Greiner, Frickenhausen, Germany) and the suspensions were filtered through a 40 µm cell strainer. An erythrocyte lysis was performed using a ready-to-use Red Blood cell lysis Buffer (Miltenyi Biotec, Bergisch Gladbach, Germany, #130–094–183) in accordance with the manufacturer’s instructions.

Cell suspensions were incubated with 4% murine Fc receptor binding inhibitor (Miltenyi Biotec, Bergisch Gladbach, Germany, #130–092–575) in phosphate buffered saline (PBS) 0.5% bovine serum albumin (BSA) and a live-death-marker (1% ZombieUV™, Biolegend, Koblenz, Germany, #423101) for 10 min on ice. Cells were stained with fluorochrome-coupled antibodies (Suppl. Tables 1 or 2) for 20 min on ice in the dark. As an internal counting standard, flow-count fluorospheres were added (Bangs Laboratories, Fishers, Indiana, USA, #7547053). Sample acquisition was performed using a FACSymphony A5SE flow cytometer (BD Bioscience, Heidelberg, Germany) and data were analyzed using FlowJo software V.10 (Tree Star). Antibodies and reagents were tested for optimal conditions before the start of the experiments. Single-color compensation to create multicolor compensation matrices was done with Comp-Beads (Biolegend, Koblenz, Germany, # 424602). Using the internal counting standard, the acquired samples were first normalized to the total sample volume and subsequently normalized either to the total mass of the dissected tumors or to the number of detected viable CD45⁺ cells. The gating strategy for the FACS analyses is indicated in Suppl. Figure 2A.

### Cell lines

B16BL6 melanoma cells were purchased from ATCC (LGC standard GmbH, Wesel, Germany) and cultured in Dulbecco´s Modified Eagle Medium (DMEM) + 4,5 g/L D-Glucose, L-Glutamine + Pyruvat (Gibco/Thermo Fisher Scientific, Dreieich, Germany, #41966–029) containing 10% fetal calf serum (FCS), penicillin/streptomycin (100U/mL each) under 5% CO_2_ humidified atmosphere. Human umbilical vein endothelial cells (HUVEC) were purchased from Thermo Fisher Scientific (Dreieich, Germany, #C0035,) and cultured in basal medium for macrovascular endothelial cells (Thermo Fisher Scientific, Dreieich, Germany, #M200500) supplemented with LVES (Thermo Fisher Scientific, Dreieich, Germany, #A1460801). HUVEC were transfected with IKKε siRNA (#s18538) or a scrambled control siRNA (#4390846) using Lipofectamine RNAiMAX (#13778100) (all Thermo Fisher Scientific, Germany). The effectiveness of the transfection was verified using qPCR analysis.

### T-cell co-culture

CD8⁺ T-cells and γδ-T-cells were isolated from murine spleens using specific isolation kits from Miltenyi according to the manufacturer’s recommendations (Bergisch Gladbach, Germany, #130–117-044 or # 30–092–125). The cells were cultured in T-cell medium (RPMI 1640 with glutamax (Gibco/Thermo Fisher Scientific, Dreieich, Germany, #61870–010), supplemented with 100 U/mL penicillin, 100 µg/mL streptomycin, 10% FCS, 1% non-essential and essential amino acids, 1% sodium pyruvate and 1% 4-(2-hydroxyethyl)−1-piperazineethanesulfonic acid (HEPES) with addition (immediately before use) of 10 ng/mL rmIL-2 (Biological Resources Branch (BRB), Developmental Therapeutics Program (DTP), Division of Cancer Treatment and Diagnosis (DCTD), National Cancer Institute (NCI, Frederick, USA)), 50 µM β-mercaptoethanol (Gibco/ThermoFisher Scientific, Dreieich, Germany, #31350–010) and mouse T-cell activator CD3/CD28 Beads (Miltenyi, Bergisch Gladbach, Germany, #130–093–627). Cultivation times before co-culture start were 2 days for CD8⁺ T-cells and 6–8 days for γδ-T-cells. Co-culture with B16BL6 cells was performed at an effector-target ratio of 10:1 (T-cells as effector cells, 5 × 10^4^ in 96-well) for 24 h without addition of IL-2 and mercaptoethanol. B16BL6 cells were seeded 24 h prior co-culture. At the endpoint, supernatants were collected for IFN-γ and granzyme B determination and cell proliferation of B16BL6 cells was analyzed via the WST-assay (Roche Diagnostics GmbH, Mannheim, Germany, #11644807001) after detaching the T-cells.

### T-cell migration

CD3 T-cells were isolated from the spleen of wildtype and IKKε KO mice using a Pan T-cell Isolation Kit II, mouse, (Miltenyi Bergisch Gladbach, Germany, #130–095–130) and directly used without prior activation with CD3/CD28 Beads. The cells were plated into cell culture inserts with 5.0 µm pore polycarbonate membrane (Corning, Kaiserslautern, Germany, #3421) into 24-well plates in medium containing 0.1% FCS. The bottom chamber below the inserts contained either 0.1% FCS medium (negative control), 10% FCS medium (positive control) or conditioned medium from B16BL6 melanoma cells cultured for 24 h without FCS. Cells were harvested 4 h later, and migrated and non-migrated cells were analyzed by FACS (antibodies see Suppl. Table 2).

### Preparation of protein lysates and nuclear extracts

Murine tumors, that were used for protein extraction, were frozen in liquid nitrogen immediately after dissection and stored at −80 °C until further processing.

Frozen tumors were homogenized in PhosphoSafe Extraction Buffer (Merck, Darmstadt, Germany, #71296) supplemented with protease inhibitor (1 mM Pefabloc SC, Carl Roth, Karlsruhe, Germany) using a tissue homogenizer (Polytron™ PT1200E, Kinematica AG, Malters, Switzerland) to generate a homogeneous suspension. Cultured cells were washed with 0.1 M PBS, scraped with a rubber policeman and collected in 1.5 mL tubes. For preparation of protein extracts, cell suspensions were briefly centrifuged at 10,000 rpm in an Eppendorf centrifuge and then the pellet was resuspended in PhosphoSafe Extraction Buffer (Merck, Darmstadt, Germany, #71296) containing protease inhibitor (1 mM Pefabloc SC, Carl Roth, Karlsruhe, Germany) and kept at room temperature for 3 min. Then, the cell lysate was centrifuged at 14,000 rpm for 30 min at 4 °C in an Eppendorf centrifuge and the supernatant was stored at −80 °C until further analysis.

Nuclear extracts were prepared from tumor tissue using the Nuclear Extract Kit (Active Motif, Biozol, Germany, # 40010) according to the manufacturer’s instructions. Briefly, the tumors were homogenized in 1 × hypotonic buffer containing protease and phosphatase inhibitors and DTT and incubated on ice for 15 min. After a centrifugation step at 850 × g for 10 min at 4 °C, the pellet was resuspended in a second hypotonic buffer and incubated on ice for 15 min followed by addition of detergent. After further centrifugation at 14,000 × g for 30 s, the supernatants including the cytoplasm fraction were collected in tubes and the resulting nuclear pellet was resuspended in complete lysis buffer containing protease inhibitors and DTT and incubated for 30 min on ice. Then, the samples were carefully vortexed for 30 s, centrifuged at 14,000 × g for 30 min at 4 °C and the supernatant containing the nuclear fraction was collected.

The protein content of all protein extracts was determined by Bradford assay and the samples were stored at −80 °C until further analysis.

### Enzyme-linked immunosorbent assays (ELISA)

Granzyme B, Cxcl1, Cxcl2 and VEGF protein levels were determined from cell and tumor protein lysates using Mouse Granzyme B ELISA Kit (Invitrogen/Thermo Fisher Scientific, Dreieich, Germany #BMS6079), Mouse CXCL1/KC Quantikine ELISA Kit (#MKC00B), Mouse CXCL2/MIP-2 DuoSet ELISA (#DY452) and Mouse VEGF Quantikine ELISA Kit (#MMVOO-1) (all R&D Systems, Minneapolis, Minnesota, USA) according to the manufacturer’s instructions. p65 transcription factor levels were analyzed from nuclear extracts of tumors, which were extracted using the Nuclear extract Kit from ActiveMotif (Biozol, Eching, Germany, #40010) (see above). p65 TransAM ELISA determining transcription factor activation was obtained from ActiveMotif and performed as recommended by the manufacturer (Biozol, Eching, Germany, #40096). The assay is suitable for assaying transcription factor binding to a p65 consensus-binding site which is coated on the surface of the wells. A primary antibody specific to an epitope on the bound and active form of the transcription factor is then used for detection of p65 protein.

### Cytometric bead array (CBA)

To measure IL-17A and IFN-γ in cell culture supernatants, murine Cytometric Bead Array Flex Sets (BD Biosciences, Heidelberg, Germany, # 562261, # 558296) were used as recommended by the manufacturer. In brief, the samples were mixed with capture beads and incubated for one hour at room temperature. Subsequently, PE detection reagent was added and incubated for further two hours. After a washing step, samples were measured in FACSFlow buffer via a FACSymphony A5 flow cytometer (BD Bioscience) and analyzed with FlowJo Software V.154 g.

### OLINK proteome analysis

Analysis of the tumor proteome was performed with proximity extension assay (PEA) technique using the Olink Target 96 Inflammation panel (Olink, Uppsala, Sweden), quantifying 92 inflammation-related proteins. All proteins analyzed are listed in Suppl. Table 3. The PEA assay employs paired antibodies conjugated to DNA oligonucleotides that come into proximity upon binding the target protein, enabling quantification via real-time polymerase chain reaction (PCR). The resulting threshold cycle (Ct) data underwent quality control using internal and external standards. Signals were processed in the Olink NPX Signature Software v2.0.2 (Olink, Uppsala, Sweden). Relative protein abundances were calculated from Ct values and reported as log_2_-transformed NPX (Normalized Protein Expression) values.

### Proteome analysis of B16BL6 cells

For proteome profiling, cell pellets of B16BL16 melanoma cells were lysed in urea lysis buffer (8 M urea, 20 mM HEPES, pH 8.0, 1 mM sodium orthovanadate, 2.5 mM sodium pyrophosphate, 1 mM beta-glycerophosphate). Protein concentrations of the lysates were determined using the 660 nm assay kit (Thermo Fisher Scientific, Dreieich, Germany) according to the manufacturer’s instructions. 10 µg protein per sample were reduced with DTT (10 mM for 1 h at 37 °C), alkylated with iodoacetamide (25 mM for 15 min at 37 °C in the dark) and digested using Lys-C (Wako/Fujifilm, Neuss, Germany) for 2 h at 37 °C in an enzyme-to-substrate ratio of 1:50 (w/w). After dilution with 20 mM HEPES (pH 8.0) to a concentration of 2 M urea, digestion was continued overnight with trypsin (Promega, Walldorf, Germany) at 37 °C and 1:50 (w/w) enzyme-to-substrate ratio. The peptide mixtures were acidified and desalted using peptide purification on the AssayMap Bravo with 5 μl C18 cartridges (Agilent, Frankfurt, Germany). Peptide samples were dried by vacuum centrifugation and then dissolved in 0.1% formic acid (FA). Peptide concentrations were determined using a fluorometric peptide assay (Thermo Fisher Scientific, Dreieich, Germany). The peptide samples were analyzed by LC–MS/MS on a Vanquish Neo UHPLC system (Thermo Fisher Scientific, Dreieich, Germany) coupled online to an Orbitrap Astral mass spectrometer (Thermo Fisher Scientific, Dreieich, Germany) in a data-independent acquisition scheme (DIA). 200 ng of peptides from each sample were concentrated and desalted on a PepMap Neo trap cartridge (Thermo Fisher Scientific, particle size 100 Å, inner diameter 300 µm, length 5 mm), followed by separation on a 25 cm Aurora Ultimate analytical column (Ion Opticks, Collingwood, Australia) using a 25 min method (18 min linear gradient) of 2% to 40% acetonitrile in 0.1% formic acid at a flow rate of 400 nl/min. Precursor ion survey scans were acquired using the Orbitrap mass analyzer with the following parameters: resolution 240,000, scan range *m/z* 380–980, automatic gain control (AGC) target 5 × 10^6, maximum injection time 10 ms, RF lens setting 40%. For fragment ion scans using the Astral mass analyzer, precursor ions were isolated for collision-induced dissociation (HCD) through each survey scan with an isolation window of *m/z* 2, resulting in 299 scan events. The normalized HCD collision energy was set to 25% and for fragment ion analysis the AGC target was 5 × 10^4 at a maximum injection time of 3 ms.

Raw DIA data were analyzed using DIA-NN (Academia version 2.3.2). The library was predicted from the Uniprot mouse reference proteome (UP000000589, downloaded 2025–03). For spectral-library searches, the predicted library was imported. The search settings included trypsin as the protease with an allowance for 2 missed cleavages. Carbamidomethylation was set as a fixed modification and oxidation of methionine was set as variable modifications. Peptide length was set as 7–30, and the precursor charge range was 1–4. The precursor range was 300–980 m/*z*, and fragment ion *m*/*z* was 150–2000. The false discovery rate (FDR) was set at 1% and MS accuracy was set to 4 for MS1 and to 10 for MS2. Match between runs was enabled, and protein inference was grouped on genes. Scoring was set as generic and machine learning utilized fast mode. Quantification utilized high precision strategy, and cross-run normalization was set as RT-dependent. IDs, RT, and IM profiling were selected for library generation. First, contaminants were removed and proteins with missing values were removed. To control for equal sample loading, intensities from each LC-MSMS run were normalized on the median of the summed-up intensities from each sample (Plubell et al. 2017). Data were further processed and analyzed using Perseus Software (v2.1.6.0) (Tyanova et al. 2016) and DAVID gene ontology analysis (https://davidbioinformatics.nih.gov/) (Huang et al. 2009a, 2009b).

### Immunofluorescence analyses

Tumor cryosections (8 µm) were thawed and post-fixed in 4% paraformaldehyde (PFA) for 10 min at room temperature. After a washing step with PBS, cells were permeabilized with PBS + 0.1% Triton X-100 (PBSTx) for 10 min. Non-specific binding sites were blocked with 3% BSA in PBS for 1.5 h and then the sections were incubated with primary antibodies in PBS + 1% BSA (HIF-1α, 1:400, Cell Signaling Technology, Boston, USA #36169; CD3, 1:100, Biolegend, Koblenz, Germany, #100202, CD31-PE, 1:600, BD Biosciences, USA, #553373) at 4 °C overnight. After 3 washing steps with PBS + 0.1% Tween 20 (PBST) for 5 min, fluorescently labelled secondary antibodies were applied for two hours at room temperature (Anti-rabbit Cy3, 1:1.200, Sigma-Aldrich, Darmstadt, Germany, #C2306 and Anti-rat Cy3, 1:1.200, Life Technologies/Thermo Fisher Scientific, Dreieich, Germany, #A10522). Background controls were stained with secondary antibody only. The sections were then washed three times with PBST for 5 min.

Subsequent DAPI staining was performed with 5 µM DAPI (Carl Roth GmbH + Co. KG, Karlsruhe, Germany, #6843.1) for 5 min at room temperature in the dark followed by washing three times with PBS for 5 min. Finally, the sections were mounted using Aqua-Poly/Mount mounting medium (Polyscience Inc., Warrington, USA, #18606–20) and a cover slip.

Microscopic images were acquired using a Zeiss Axio Observer Z1 inverted fluorescence microscope equipped with an HXP 120 light source, an AxioCam ERc camera and ZEN software (Zeiss, Oberkochen, Germany). Densitometric analyses of the stain were performed with ImageJ software (Version 1.54 g) after standardized background subtraction and threshold normalization.

### Multiplex immunofluorescence analysis

Paraffin-embedded primary human melanoma tissue samples provided by the University Cancer Center (UCT) Frankfurt were cut into 3 µm sections. Staining and analysis were performed with the Opal Automation Multiplex IHC Detection Kits (Akoya Biosciences, Boston, USA) to the manufacturer’s instructions. The following primary antibodies and Opals were used for staining: IKKε (1:100, Cell Signaling Technology, Boston, USA, #2905) with Opal 690, CD3 (1:500, Cell Signaling Technology, Boston, USA, #85061S) with Opal 520, CD4 (1:100. Abcam, Cambridge, UK, #ab133616) with Opal 570, CD8 (1:100, DAKO/Agilent Technologies, Santa Clara, USA, #M710301-2) with Opal 480, γδ-TCR (1:100, Santa Cruz Biotechnology, Dallas, USA, #sc-100289) with Opal 620, S100 (1:150, Abcam, Cambridge, UK, #ab4066) with Opal 780. Slides were imaged with Vectra3 Polaris automated imaging system (Akoya Biosciences) and images were analyzed using inForm2.0 Software (Akoya Biosciences). Based on S100 expression, tissue was segmented into S100-positive (tumor) and S100-negative regions. Two independent analysis algorithms were established for cell phenotyping. One was used to identify and classify tumor cells (S100⁺), T cells (CD3⁺), and IKKε-positive cells within the defined cell types. The second algorithm was designed for the detailed characterization of T-cell subsets, allowing the differentiation of CD4⁺, CD8⁺, and γδ-T-cells. Cell segmentation was performed based on DAPI-stained nuclei, whereas marker expression was quantified in a cell type-specific manner. Cells were classified according to their respective marker expression profiles. T-cell subsets were further classified according to the expression of CD4, CD8, or γδ-TCR within the CD3⁺ T-cell population (the staining scheme is shown in Suppl. Figure 1A). To ensure reproducibility, the algorithms were trained using representative image datasets and subsequently applied to all samples using identical analysis parameters. Depending on tissue size, three to eight representative fields of view were analyzed per patient sample. Following automated image analysis, all fields of view underwent manual quality control. Individual fields of view or entire tissue samples were excluded if they exhibited poor staining quality, staining failure of the respective marker, tissue-folding artifacts, excessive background staining, or insufficient image focus. Only fields of view that fulfilled the predefined quality criteria were included in the quantitative analysis.

### Tube formation assay

The tube formation assays were performed using Ibidi µ-plates (Ibidi, Gräfelfing, Germany, #81506). The inner wells of the plates were coated with 13 µl of Matrigel (Corning, Kaiserslautern, Germany, # 356231) which were allowed to polymerize for 30 min. Then, 10.000 HUVEC (naïve or treated with IKKε-siRNA or scrambled control siRNA)/well in endothelial cell basal medium supplemented with LVES were placed on the top of the Matrigel in a volume of 50 µl. The formation of a tube network was observed over a period of 24 h. Photographs were taken at time point 4 h since a stable and complete network was developed at that time-point. The analyses of the network were performed with ImageJ software (Version 1.54 g) equipped with an angiogenesis plug-in.

### Spheroid sprouting assay

The spheroid assay was performed with HUVEC treated with and without VEGF-A (Med Express/Biozol, Eching, Germany, #HY-P7110A) or conditioned medium from B16BL6 cells. 80.000 HUVEC were suspended in 4 mL HUVEC medium and 1 mL Methylcellulose solution (12 g/L), which increases viscosity. 25 µl drops of the cell suspension were then distributed on a 10 cm square Petri dish using a 12-channel pipette. Drops were incubated upside down at 37 °C for 24 h to allow spheroid formation. Spheroids were then collected with sterile PBS and centrifuged at 1,200 rpm for 5 min without brake. Afterwards, the spheroids were resuspended in Methylcellulose solution supplemented with 20% FCS and embedded in a collagen matrix (Corning/Merck, Darmstadt, Germany# CLS354236), which was allowed to polymerize for 30 min at 37 °C. After polymerization, spheroids were stimulated with either 100 µl VEGF-A supplemented medium (end concentration 30 ng/mL) or 100 µl basal medium for 24 h at 37 °C. Images were taken with a transmitted-light microscope ZEISS Axio Observer equipped with the Axiocam Erc 5 s camera system and analyzed using ZEN software (Zeiss, Oberkochen, Germany) as well as ImageJ (Version 1.54 g). The resulting sprouts were quantified by determination of the cumulative sprout length (CSL).

### Western Blot analysis

Proteins (20 µg) were separated electrophoretically by 10% SDS-PAGE and then transferred onto nitrocellulose membranes by wet-blotting. To control the quality of the transfer, all Blots were stained with Ponceau red solution. Membranes were blocked for 60 min at room temperature in Odyssey blocking reagent (LI-COR Biosciences, Bad Homburg, Germany, #927–700001) diluted 1:2 in 0.1 M PBS, pH 7.4. Afterwards, the Blots were incubated overnight at 4 °C with primary antibody against MMP-9 (1:250) R&D Systems, Minneapolis, Minnesota, USA, #AF909), VEGF (1:250, Invitrogen/Thermo Fisher Scientific, #MA5-13182), p65 (1:250, Cell Signaling Technology, Boston, USA, #8242S), IKKε (1:250, Cell Signaling Technology, Boston, USA, #3416) in Odyssey blocking reagent diluted 1:2 in 0.1% Tween 20 in 0.1 M PBS. After washing three times with 0.1% Tween 20 in 0.1 M PBS, the Blots were incubated for 60 min with an IRDye 700-conjugated secondary antibody (Molecular Probes, Invitrogen/Thermo Fisher Scientific, 1:10.000 in blocking buffer diluted 1:2 in 0.1% Tween 20 in 0.1 M PBS). After rinsing in 0.1% Tween 20 in 0.1 M PBS, protein-antibody complexes were detected with the Odyssey Infrared Imaging System (LI-COR Biosciences). β-actin (37 kDa) (1:2.000, Sigma, Merck, Darmstadt, Germany, # A5441) was used as loading control for cytosolic extracts, histone H3 (1:250, Cell Signaling Technology, Boston, USA, #4620S) for nuclear extracts. Densitometric analysis of the Blots was performed with Image Studio Lite Software V6.0 (LI-COR, Biosciences, Bad Homburg, Germany).

### Zymography assay

Proteins (20 µg) were separated by electrophoresis on 10% SDS–polyacrylamide gels containing 1 mg/mL gelatin as a matrix metalloproteinase substrate, without prior boiling of the samples. After electrophoresis, gels were incubated in renaturation buffer (25% Triton-X-100 in H₂O) and subsequently equilibrated for 30 min at room temperature in developing buffer before being incubated overnight at 37 °C. Gels were then stained with Coomassie Blue for 30 min in the dark and destained for an additional 30 min. Finally, gels were scanned using the Odyssey Infrared Imaging System (LI-COR Biosciences), and band intensities were quantified with Image Studio Lite Software V6.0 (LI-COR Biosciences, Bad Homburg, Germany).

### Quantitative real-time polymerase chain reaction (qRT-PCR)

RNA-isolation from harvested HUVEC was performed using the Qiagen RNeasy Mini Kit (Qiagen, Hilden, Germany, #74106) according to the manufacturer´s instructions. Two hundred nanograms of total RNA were used for the reverse transcription which was performed using a Verso cDNA Kit (Thermo Fisher Scientific Dreieich, Germany, #AB1453B). Twenty nanograms of RNA equivalent were subjected to real-time PCR in an Applied Biosystems sequence detection system QuantStudio™ 5 (Thermo Fisher Scientific, Dreieich, Germany) using ORA SEE qPCR Green as fluorescence staining (HighQu GmbH, Kraichtal, Germany, #QPD0550c1). Expression of IKKε was determined and normalized to GAPDH as housekeeping gene. Relative quantitative levels of samples were determined by standard 2^−ddCt^ calculations and expressed as fold change of a reference control sample (cells treated with NC).

The following gen-specific primers were used:

- IKKε (human): FW 5´– TAG TCA CAC ACG GCA AGA GG – 3´
  RV 5´– TAG CTC TTC CAG GAG CTT GC – 3´
- GAPDH (human): FW 5′– ACA ACT TTG GTA TCG TGG AAG G – 3′
  FW 5′– ACA ACT TTG GTA TCG TGG AAG G – 3′

### Data analysis

Statistical evaluation was done with Graph Pad Prism 11 for Windows. Data are presented as mean ± SEM. All data sets were checked for normality and lognormality by applying Shapiro–Wilk test. Data were either compared by univariate analysis of variance (ANOVA) with subsequent t-tests employing a Bonferroni α-correction or Dunnett’s-correction for multiple comparisons, by repeated-measures two-way ANOVA or by Student´s t-test. For all tests, a probability value p < 0.05 was considered as statistically significant. The two tumors from each mouse were averaged, and the resulting per-mouse mean was used for statistical analysis. For the T-cell–depletion groups, tumor growth accelerated so rapidly that mice had to be euthanized upon reaching ethical endpoints. Therefore, in addition to showing tumor growth as determined until d16, few values at later time points were derived mathematically. For this purpose an exponential growth model was used (V(t) = V0 * e ^r*t^). To avoid implausibly extrapolated tumor volumes, a maximum cutoff of 10 cm^3^ was defined for the mathematically modeling. Tumors with calculated volumes exceeding this value were excluded from further analysis. The survival analysis of human melanoma patients was recalculated using the raw data available from the Human Protein Atlas. With X-tile software (Yale University, Version 3.6.1) (Camp et al. 2004), an X-tile optimization to determine an objective expression cut-off was performed. Furthermore, multivariable Cox proportional hazards regression was calculated to evaluate whether IKKε expression provides independent prognostic information for overall survival. The model was adjusted for sex, age, tumor stage and survival time.

## Results

### IKKε is expressed in tumor cells and CD4+, CD8⁺ and γδ-T-cells of human primary melanoma

**Fig. 1:**
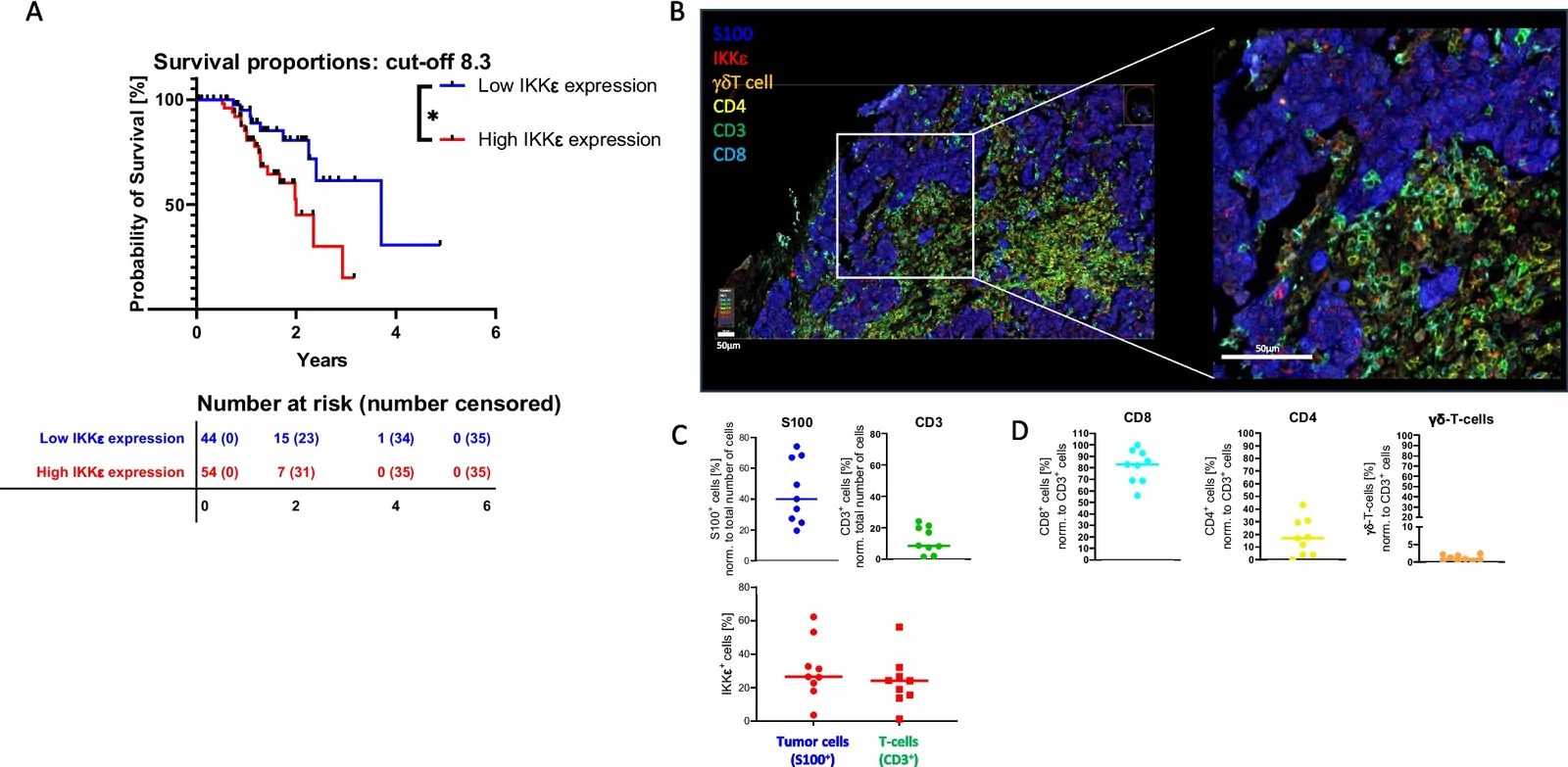
Epidemiology and expression of IKKε in human skin cancer. **A** Survival proportions of skin cancer patients in relation to IKKε expression levels, adapted from the Human Protein Atlas (https://www.proteinatlas.org/).The cut-off IKKε expression was calculated by X-tile optimization. *P < 0.05, significant difference between low and high IKKε expression. **B** Multiplex immunofluorescence analysis showing protein expression of IKKε (red), tumor (S100, blue) and T-cells (CD3^+^, green; CD4^+^, yellow; CD8⁺, turquoise; γδ-T-cells, orange) in human primary melanoma (representative picture from *n* = 9 patient samples), Scale Bars: 50 µm. **C** Quantitative analysis of tumor cells (S100⁺) and CD3⁺ T cells, including colocalization of IKKε expression within tumor cells and CD3⁺ T cells. Values are presented as the percentage of S100⁺ or CD3⁺ cells normalized to the total cell count, or as the proportion of IKKε-positive cells relative to the total number of analyzed cells within the cell type (tumor cells or CD3⁺ T cells), respectively. **D** Quantitative analysis of T-cell subsets in human melanoma samples. Values are shown as percentages relative to the CD3⁺ T-cell population. Horizontal lines indicate the median. Each dot represents one patient sample (*n* = 9). For each sample, 3–8 representative fields of view were analyzed

Data from the human protein atlas (https://www.proteinatlas.org/; https://v18.proteinatlas.org/ENSG00000263528-IKBKE/pathology/tissue/melanoma) (Camp et al. 2004) indicate that IKKε levels in the tumor have an impact on prognosis and survival of melanoma patients with high IKKε expression being associated with poorer overall survival (Hazard Ratio 2.73, 95% CI 1.16–6.43, *p* = 0.021) (Fig. 1A, Suppl. Table 4). Although the difference between high and low IKKε expression reaches statistical significance, the relatively high censoring rate of the open study limits the interpretation of the data. A multiepitope immunologic staining screen in primary human melanoma slices showed IKKε protein expression in highly proliferating tumor cells (S100 +) as well as different types of T-cells. The immunofluorescence images revealed the expression of IKKε in CD3^+^, CD4^+^, CD8⁺ and γδ-T-cells (Fig. 1B). A quantitative analysis of the images indicated that T-cells accounted for median of 8.72% (range: 1.5-24.2%) and tumor cells for a median of 40.1% (range 19.6–74.14%) of all cells in the tumor. IKKε is expressed at comparable levels in tumor cells and T-cells with median expression levels of 26.4% (range: 3.5-62,3%) in tumor cells and 24.0% (range: 1.1–56.25%) in T-cells (Fig. 1C). Within the T-cell compartment CD8⁺ T-cells represented the predominant population (median 82.9%, range: 55.9-99.7%). CD4^+^ T-cells accounted for median 16.88% (range: 0,2—43,5%) and γδ-T-cell constituted a minor fraction with a median frequency of 0.9% (range:0.1—2.5%) (Fig. 1D). These results further support a role for IKKε in both tumor cells and the tumor microenvironment in melanoma.

### Deletion of IKKε reduces tumor growth in mice

**Fig. 2:**
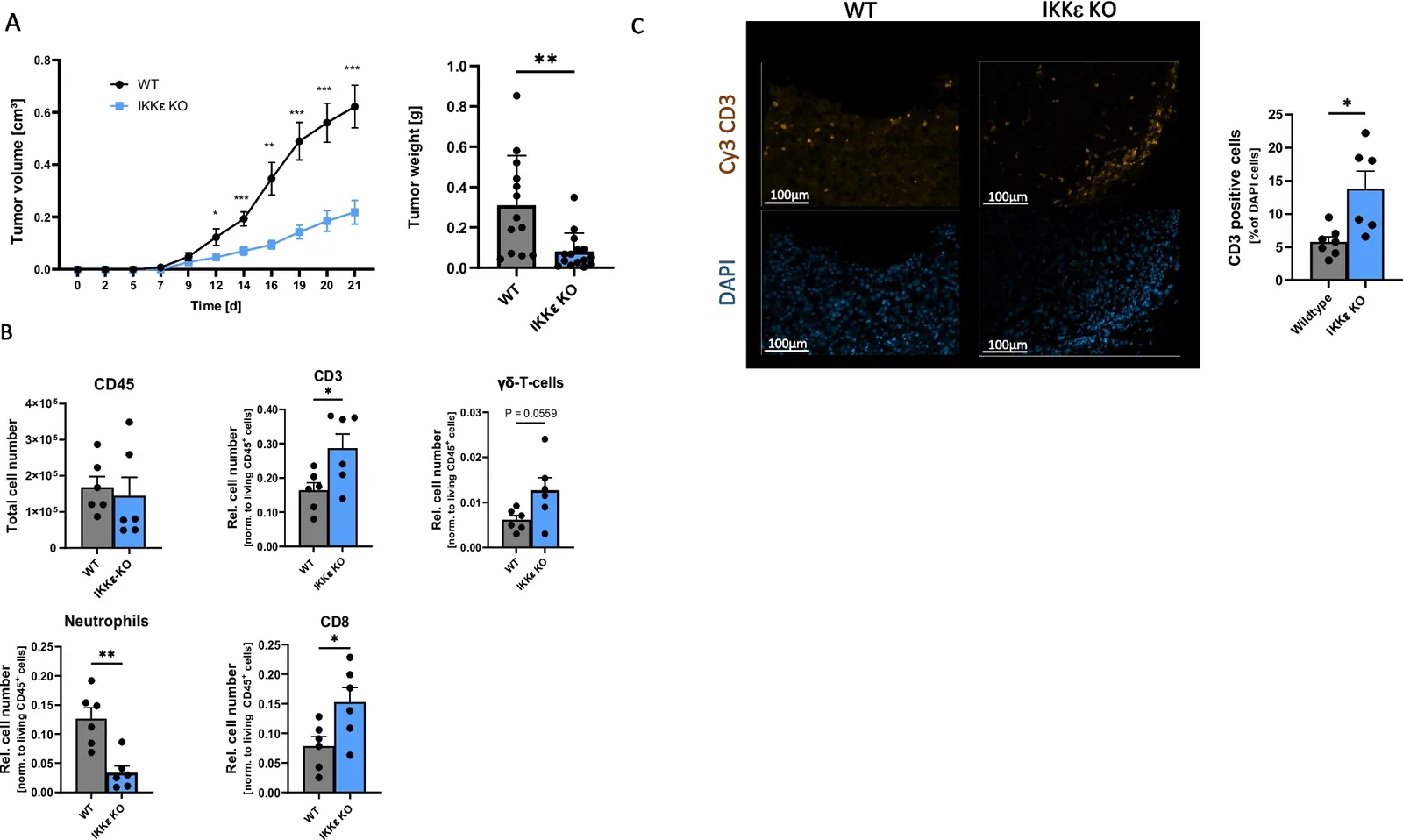
Tumor growth, immune cell composition and CD3 T-cell immunofluorescence. **A** Tumor growth in wildtype (WT) and IKKε KO mice over a period of 21 days and tumor masses at the end of the experiment. WT: *n* = 13 mice, IKKε KO: *n* = 15 mice, Two-way Anova, Tukey´s multiple comparisons test; tumor mass, Mann Whitney test. **B** Immune cell numbers in the tumors as determined by flow cytometry. Cell counts were normalized to the number of living CD45^+^ immune cells. *n* = 6/group; Student´s t-test. **C** Immunofluorescence analysis of CD3-positive cells in the tumor. Nuclei were stained with DAPI and CD3 signals normalized to the cell number. Scale Bar represents 100 µm. Representative figure from WT *n* = 7, and IKKε KO *n* = 6, the diagram shows the quantitative analysis of all tumors analyzed. Student´s t-test. *P < 0.05, **P < 0.01, ***P < 0.001

B16BL6 melanoma cells were injected subcutaneously into both flanks of wildtype and IKKε KO mice and the tumor growth was observed over a period of up to 21 days. The results demonstrated that tumors first appeared on day 7 in wildtype mice and on day 9 in IKKε KO mice. In wildtype mice, the tumors exhibited continuous growth until the termination of the experiment on day 21, at which they attained an average volume of approximately 0.7 cm^3^. In the IKKε KO mice, the progression of the tumors was significantly inhibited, with tumor volumes reaching a maximum size of 0.2 cm^3^ by the end of the experiment. This phenomenon is also reflected in the final tumor mass, which was significantly lower in mice with an IKKε KO (Fig. 2A). To examine potential differences in the TME, FACS analyses with an immune cell antibody panel were performed and revealed an increase in CD3-positive cells in tumors of IKKε KO in comparison to wildtype mice which could be attributed to a significant ⁓2-fold rise in CD8⁺ and a tendential, non-significant increase of γδ-T-cells. In addition to changes in T-cell levels, a decreased level of neutrophils in the tumors of IKKε KO as compared to wildtype mice was observed. CD4^+^ T-cells, T_regs_, macrophages, dendritic cells and NK-cells were not altered (Fig. 2B, Suppl. Figure 1). To gain more insight into the spatial distribution of T-cells within the tumor, immunofluorescence experiments were applied which showed a ⁓3-fold increased presence of CD3-positive T-cells in tumors derived from IKKε KO mice. The morphology of the staining indicates an invasion and accumulation of T-cells in the peripheral regions of the tumors (Fig. 2C). The quantitative divergence in T-cell levels between FACS and immunofluorescence assays can be attributed to the distinct regional staining in the sections compared with the analysis of the entire tumor mass.

### CD8⁺ T-cells from IKKε KO mice show higher activity than wildtype CD8⁺ T-cells

**Fig. 3:**
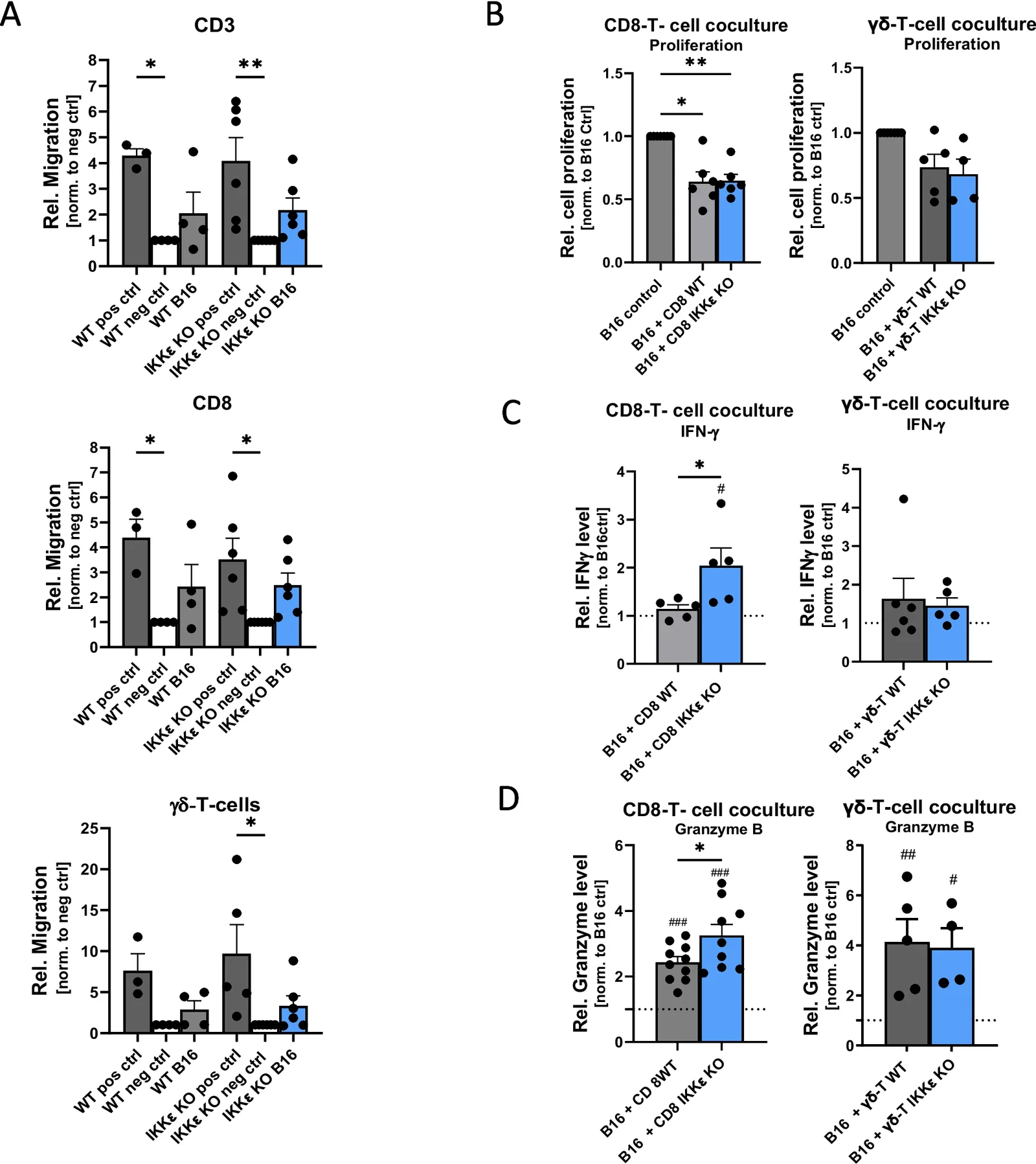
T-cell migration and activity. **A** T-cell migration towards a FCS gradient or conditioned medium from B16BL6 melanoma cells. Pan T-cells were isolated from the spleens of wildtype (WT) and IKKε KO mice and plated into cell culture insert filled with T-cell medium containing 0.1% FCS. The bottom chamber was filled either with medium containing 0.1% FCS (neg ctrl), 10% FCS (pos ctrl) or conditioned medium from B16BL6 cells without FCS (B16). Cell migration of different T-cell subtypes to the lower channel was determined by FACS analysis. Cell numbers were normalized to counting beads and negative controls. *n* = 3–6. One-way ANOVA with Tukey´s multiple comparisons test, *P < 0.05, **P < 0.01. **B**—**D** Effects of co-culture of B16BL6 melanoma cells with CD8⁺ and γδ-T-cells, respectively, derived from the spleens of wildtype (WT) and IKKε KO mice. **B** Cell proliferation determined by WST assay, B16BL6 monocultures were set as 1 for better comparison. One-way ANOVA with Tukey´s multiple comparisons test, *P < 0.05, **P < 0.01 significant difference in comparison to B16BL6 monoculture (**C**) IFN-γ levels analyzed by cytokine bead array (CBA) and (**D**) granzyme B release determined by ELISA. *n* = 4–10/group. **C** and **D** The T-cell monocultures of wildtype and IKKε knock-out mice were set as 1 for better comparison. Student´s t-test, *P < 0.05, significant difference in comparison to wildtype, #*P* < 0.05, ##*P* < 0.01, ###*P* < 0.001 significant difference in comparison to T-cell monocultures

To examine whether the enhanced tumor-killing capacity of T-cells in the IKKε KO mice is attributable to increased T-cell activity or to enhanced migration, resulting in the higher number of T-cells in the tumors, cell culture experiments were performed. Migration of primary T-cells isolated from the spleens of wildtype and IKKε KO mice was increased in positive controls with 10% FCS and conditioned medium from B16BL6 cells in comparison to the negative controls with 0.1% FCS medium in the lower chamber. However, no difference was observed between the genotypes (Fig. 3A). Co-cultures of B16BL6 with either CD8⁺- or γδ-T-cells from wildtype and IKKε KO mice, respectively, demonstrated the expected reduction of melanoma cell proliferation in comparison with B16BL6 monoculture. However, this effect was similar in wildtype and IKKε KO T-cells in co-culture with B16BL6 (Fig. 3B). The assessment of T-cell activity markers interferon-γ (IFN- γ) and granzyme B in co-culture revealed significantly enhanced activation of CD8⁺ cells from IKKε KO mice (Fig. 3C). Conversely, no differences between the genotypes were observed for γδ-T-cells (Fig. 3D).

**Fig. 4:**
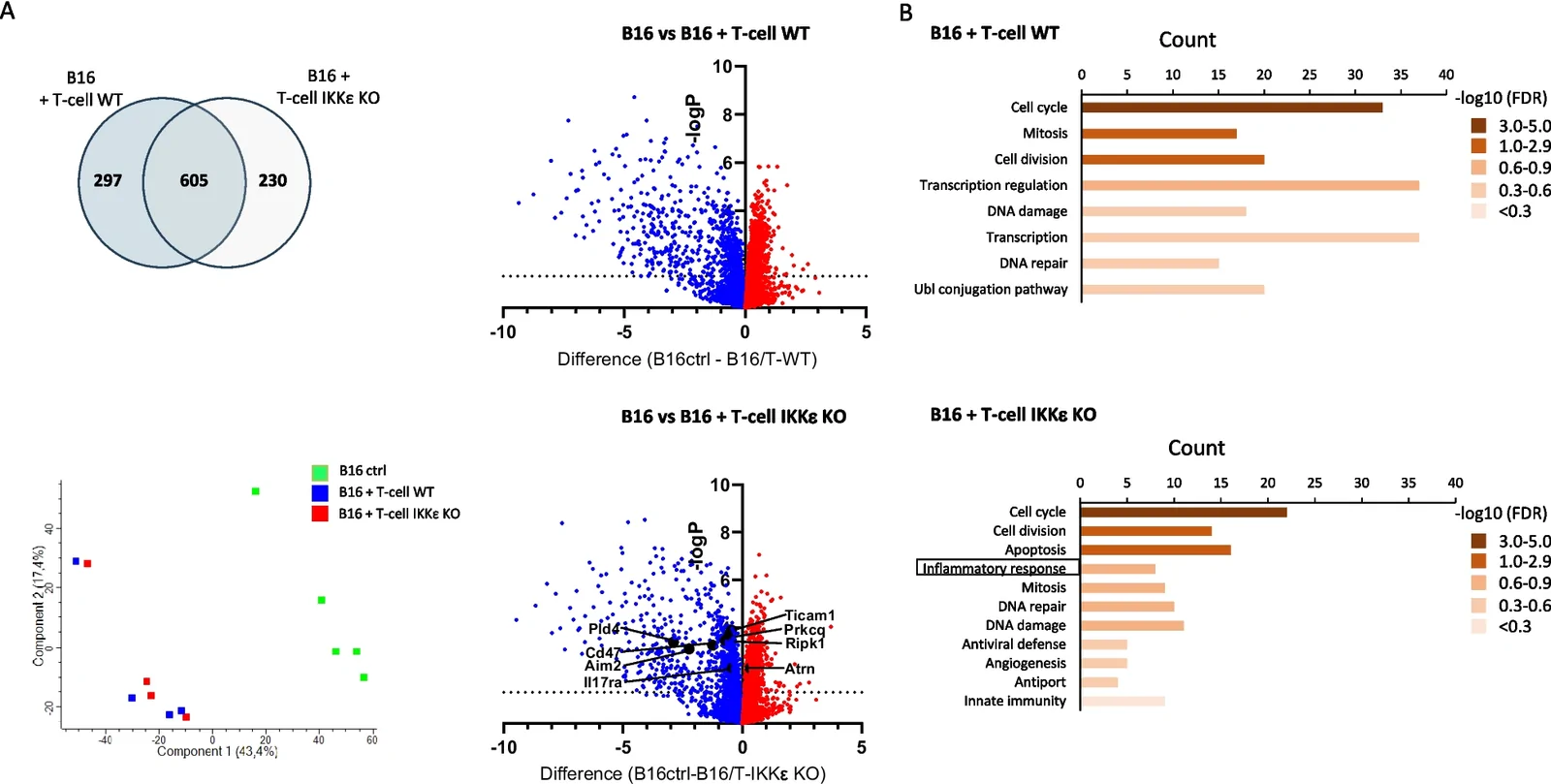
Proteomic analysis of B16BL6 cells with and without co-culture with WT or IKKε KO CD8⁺ T-cells. B16BL6 were either cultured as monoculture or as co-culture with CD8⁺ T-cells from wildtype and IKKε KO mice. Resulting protein lysates were subjected to proteome analysis by LC-MSMS. **A** Venn diagram, PCA and Volcano plots showing apparent differences between mono- and co-culture as well as proteins regulated by wildtype or IKKε KO T-cells, respectively. **B** Gene ontology analysis of differentially regulated proteins. Proteins involved in the significant regulation of the inflammatory response in co-culture of B16BL6 with IKKε KO T-cells are indicated in the respective Volcano plot. *n* = 5 B16BL6 monoculture-and *n* = 4 for B16BL6-T-cell co-cultures

From these data, it could be concluded that within the T-cell population CD8⁺ T-cells play the most important role in the IKKε-mediated regulation of melanoma tumor growth. Therefore, a proteomic analysis was performed with protein lysates of B16BL6 cells with and without co-culture with CD8⁺ T-cells derived from wildtype or IKKε KO mice, respectively. On average 9100 proteins in B16BL6 cells were identified by LC-MSMS analysis per sample with approximately 6800 proteins that could be quantified in at least 70% of the samples. The results showed strong changes in the proteome of B16BL6 cells in monoculture in comparison with B16BL6 cells in T-cell co-culture as shown in the principal component analysis. 1132 proteins were significantly regulated by both wildtype and IKKε KO T-cell co-culture. Direct comparison of the cocultures revealed no significant differences (Suppl. Figure 2); however, 605 proteins were modulated similarly in both co-cultures while 297 proteins were regulated in wildtype co-culture and 230 proteins in IKKε KO co-culture only (Fig. 4A). Gene ontology analyses of these markers showed a marked regulation of proteins involved in cell cycle and transcriptional processes in both genotypes. In co-cultures with IKKε KO T-cells there were additional regulation patterns in inflammatory processes, in which most proinflammatory proteins were upregulated (Fig. 4B), indicating an activation of the immune response.

### Impact of CD8⁺ T-cell depletion in vivo

**Fig. 5:**
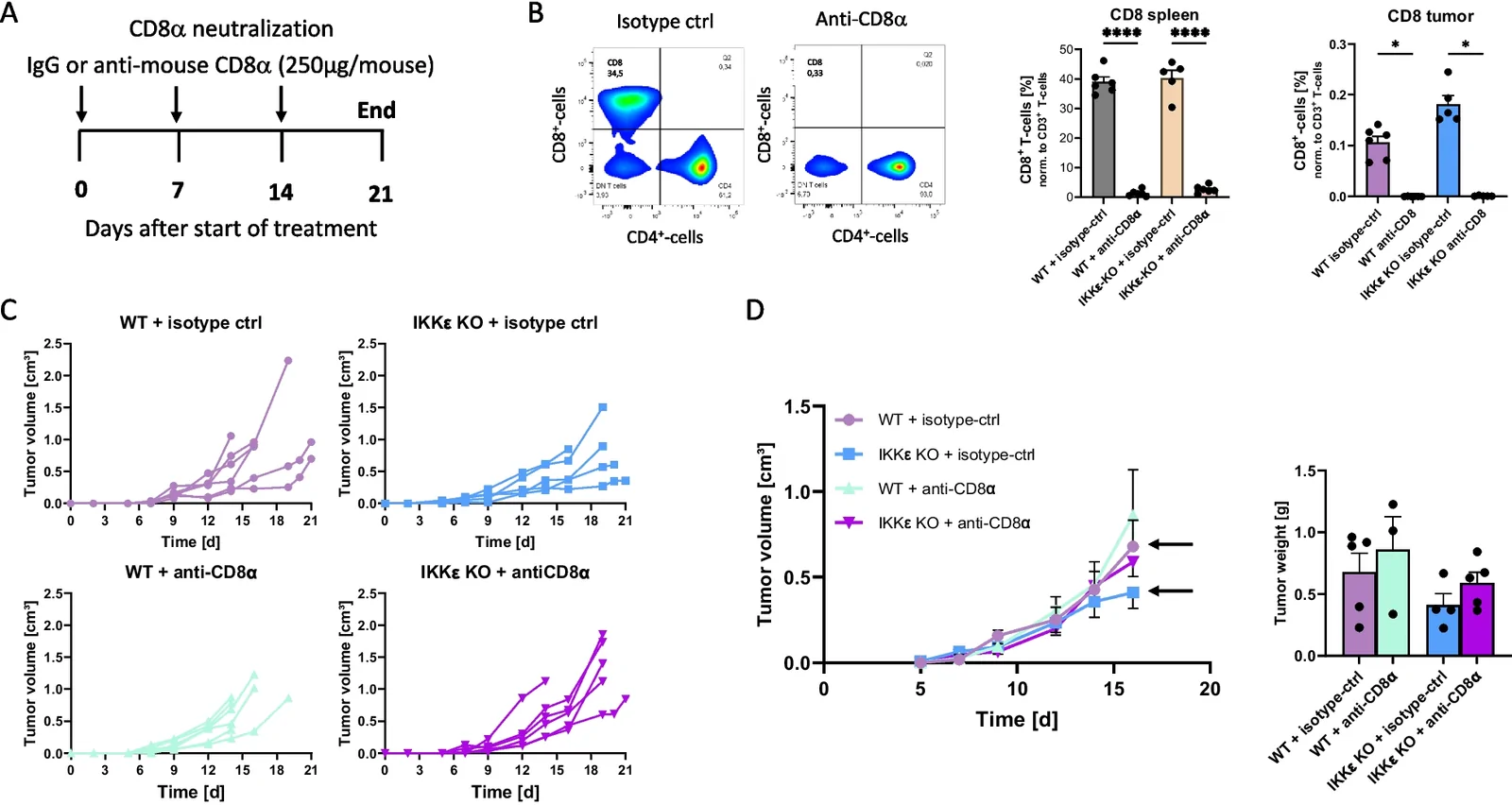
Tumor growth after depletion of CD8⁺ T-cells. **A** CD8⁺ T-cells were depleted in wildtype and IKKε KO mice by intraperitoneal injection of anti-mouse CD8α as indicated. IgG injection served as isotype control. **B** Successful depletion was verified by FACS analysis of spleen and tumor samples. The plot shows a representative example depicting CD4, CD8, and double-negative (DN; CD3⁺, CD4⁻, CD8⁻) T-cell populations. **C** Tumor growth kinetics for each individual mouse over a 21-day period. **D** Left panel: tumor growth kinetics including only mice that survived at least until day 16. Two-way ANOVA with Tukey’s multiple-comparisons test was applied. Arrows indicate tumor volumes from isotype-control–treated mice. Right panel: tumor mass, analyzed by one-way ANOVA with Tukey’s post hoc test. Group sizes: all mice: WT + isotype control, *n* = 6; WT + anti-CD8α, *n* = 6; IKKε-KO + isotype control, *n* = 5; IKKε-KO + anti-CD8α, *n* = 6.; mice that survived until day 16: WT + isotype control, *n* = 5; WT + anti-CD8α, *n* = 3 mice; IKKε-KO + isotype control, *n* = 4; IKKε-KO + anti-CD8α, *n* = 5

To further examine the impact of CD8⁺ T-cells on the reduced tumor growth in IKKε KO mice, in vivo depletion experiments were performed. For this purpose, depleting antibodies for CD8⁺ were injected intraperitoneally into wildtype and IKKε KO mice at the time of tumor inoculation and then once weekly. Control animals received the respective IgG control. The successful depletion of CD8⁺ T cells after antibody injection, compared with the isotype control, was confirmed in spleen and tumor preparations collected at the end of the experiment (Fig. 5B). In these experiments, tumors developed earlier and progressed more rapidly, particularly in wildtype mice treated with anti-CD8α. As a consequence, several mice had to be sacrificed before day 16 due to termination criteria (Fig. 5C). The mice that survived until day 16 showed results consistent with our previous untreated experiments: wildtype mice exhibited stronger tumor growth than IKKε-knockout mice after treatment with control IgG. Injection of the CD8⁺ antibody increased tumor growth in both genotypes, resulting in tumor volumes in IKKε-knockout mice that were comparable to IgG-treated wildtype mice (Fig. 5D). Mathematical extrapolation of the missing data points up to day 21 illustrates this trend more clearly (Suppl. Figure 3), suggesting that the elevated CD8⁺-cell levels in IKKε-knockout mice contribute to the suppression of tumor progression. However, it should be noted that no statistically significant differences were obtained due to high inter-individual variability in the data.

### IKKε promotes tumor angiogenesis

**Fig. 6:**
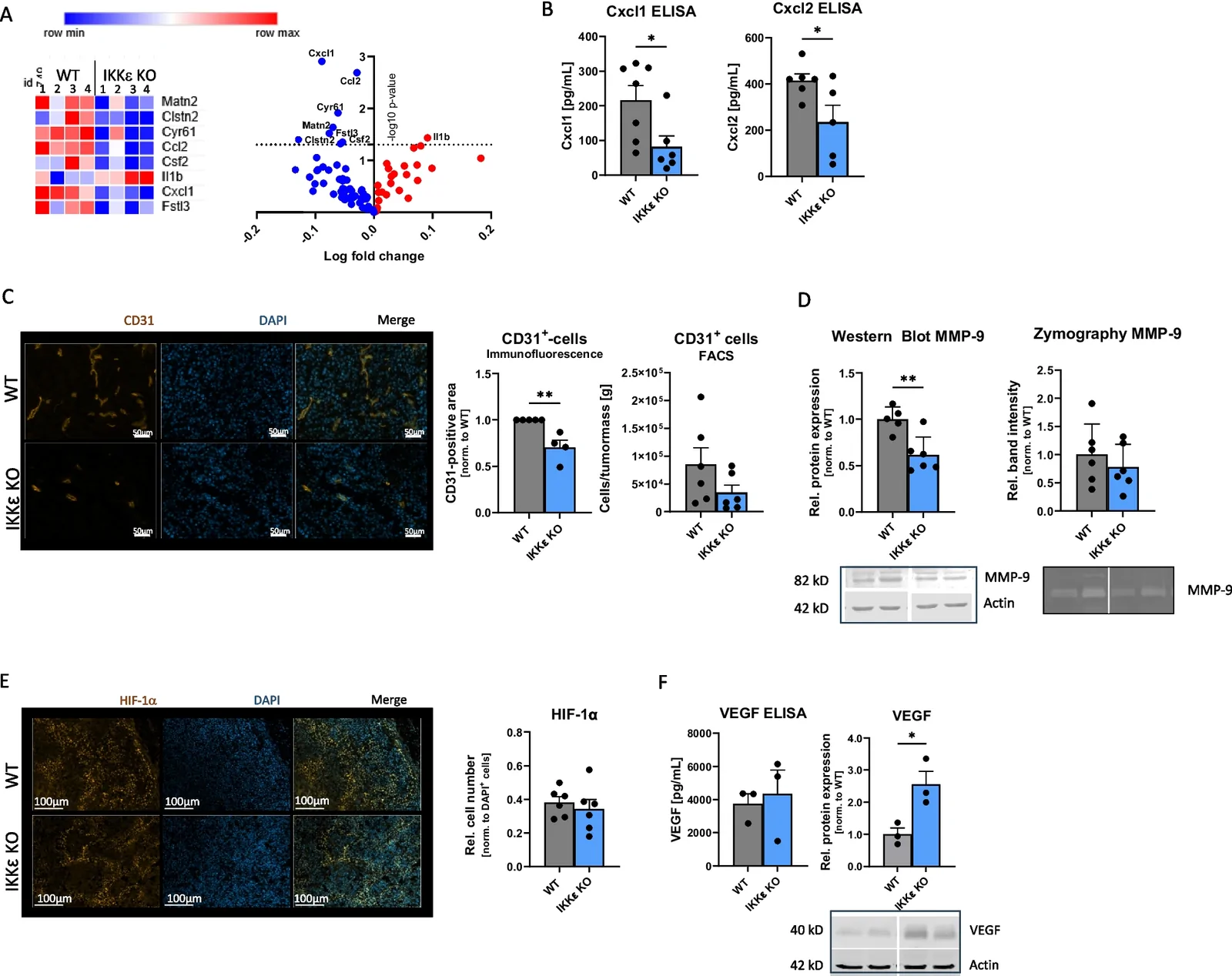
Regulation of cytokines and angiogenesis markers in the tumor. **A** OLINK proteomic analysis of inflammatory cytokines and chemokines in the tumors of wildtype and IKKε KO mice at the end of the experiment. Heat map and Volcano Plot of significantly different regulations. *n* = 4/group (**B**) Cxcl1 and Cxcl2 ELISA. Cxcl1: WT *n* = 8, IKKε-KO *n* = 6; Cxcl2: WT *n* = 6, IKKε-KO *n* = 5. **C** Determination of CD31-positive cells as vessel markers in the tumors. Left panel: FACS analysis, *n* = 6 per group. Right panel: immunofluorescence images and quantitative analysis, *n* = 4–5 tumors per group; 3–5 areas per tumor were evaluated. CD31-positive areas were normalized to wildtype controls to facilitate comparison. **D** MMP-9 expression as assessed by Western blot and zymography, *n* = 5–6 per group. **E** HIF-1α expression as determined by immunofluorescence, the pictures show on representative stain from 6 independent analyses, the diagram shows the densitometric analysis of all experiments, scale bar represents 100 µm. **F** VEGF expression analyzed by ELISA and Western Blot, n = 3/group. Student´s t-test, *P < 0.05, **P < 0.01

Since CD8^+^ T-cell depletion only partially explained the effects of IKKε in tumor progression, we performed an OLINK targeted proteomic analysis with tumor lysates focusing on inflammatory cytokines and chemokines to identify molecular mechanisms that may underlie the reduced tumor growth observed in IKKε-deficient mice. The data revealed a marked reduction in C–C motif chemokine ligand 2 (Ccl2), C-X-C motif chemokine ligand (Cxcl)1, and cysteine-rich angiogenic inducer 61 (Cyr61) in tumors from IKKε KO mice (Fig. 6A). Because the downregulated proteins are well-established mediators of angiogenesis (Koga et al. 2008; Dhawan and Richmond 2002; Kular et al. 2011), these findings suggested that IKKε inhibition may impair tumor-induced neovascularization. Subsequent experiments confirmed the decrease in Cxcl1 by ELISA, and additionally demonstrated reduced levels of Cxcl2, another agonist of the CXC-motif-chemokine receptor 2 (Cxcr2) (Fig. 6B). Further evidence for diminished angiogenesis in tumors of IKKε-deficient mice included reduced matrix metalloproteinase-9 (MMP-9) levels revealed by Western blot and zymography analyses and a lower abundance of CD31^+^ endothelial cells as determined by FACS and immunofluorescence, respectively (Fig. 6C, D). In contrast, hypoxia-inducible factor 1-α (HIF-1α) levels remained unchanged, whereas vascular endothelial growth factor (VEGF) expression was slightly increased in ELISA and significantly elevated in Western Blot analyses (Fig. 6E, F).

**Fig. 7:**
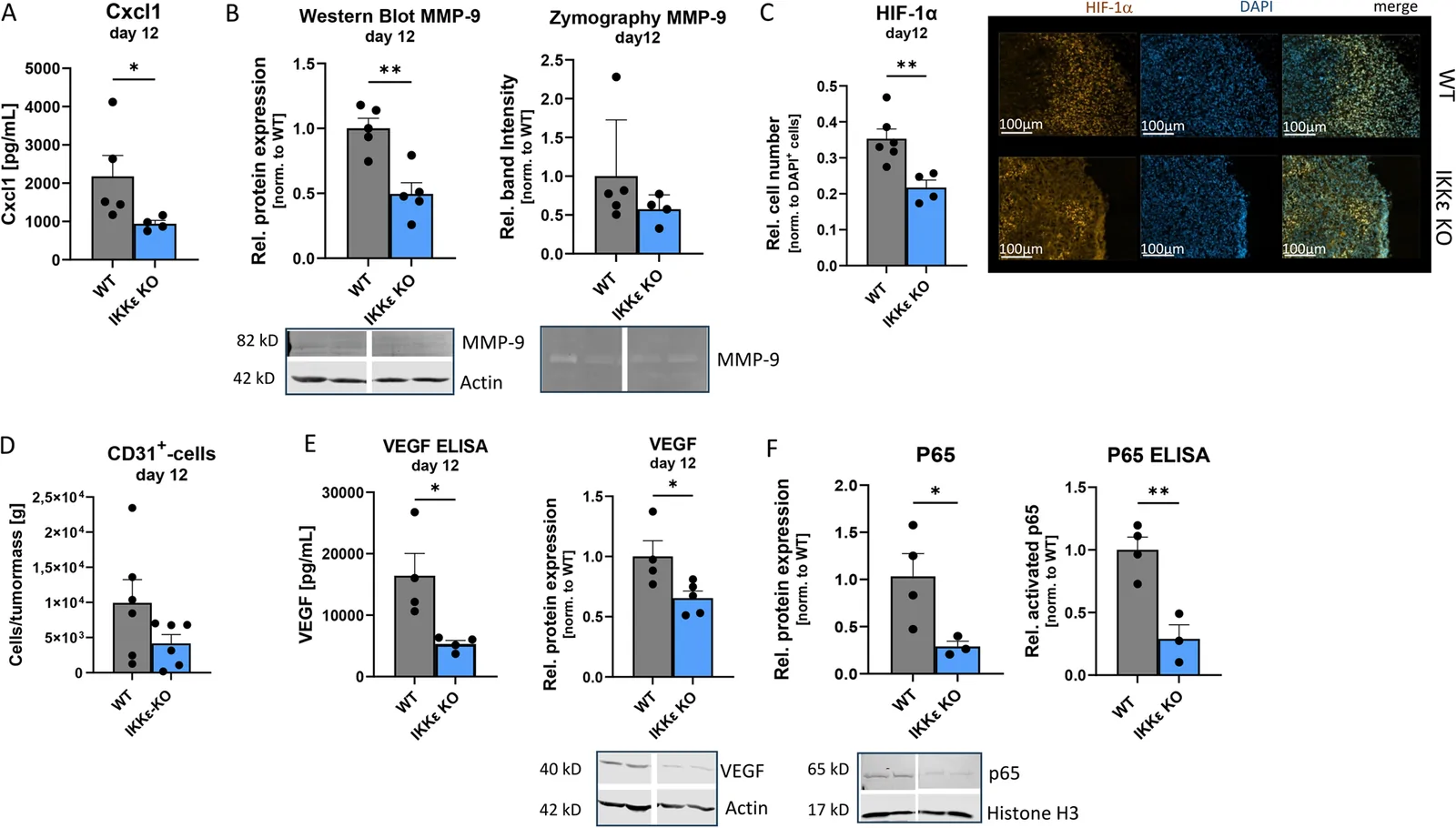
Early regulation of cytokines and angiogenesis markers in the tumor. **A** Cxcl1 ELISA, (**B**) MMP-9 expression (Western Blot and zymography), (**C**) HIF-1α expression as determined by immunofluorescence. The pictures show a representative stain from 4–6 independent analyses, the diagram shows the densitometric analysis of all experiments, scale bar represents 100 µm. **D** FACS analysis of CD31.^+^-cells as vessel markers in the tumors, (*n* = 6/group). **E** VEGF expression analyzed by ELISA and Western Blot, (**F**) p65 expression and activity in nuclear extracts as determined by Western Blot and transcription factor ELISA. *n* = 3–5/group. Student´s t-test, *P < 0.05, **P < 0.01

To exclude the possibility that these alterations were secondary to differences in tumor size between wildtype and IKKε KO mice, we additionally analyzed tumors at an early time point (day 12 after inoculation), when genotype-dependent growth differences had only begun to emerge. Even at this stage, Cxcl1 and MMP-9 were significantly downregulated, and both HIF-1α and VEGF were reduced, supporting a primary effect of IKKε loss on angiogenic signaling (Fig. 7A-D). Given that IKKε is a regulator of NF-κB signaling and many of the affected proteins are NF-κB targets, we assessed NF-κB p65 expression and activity by analyzing nuclear lysates of the tumors. As expected, p65 activity was significantly reduced in tumor nuclear extracts from IKKε KO mice (Fig. 7E).

**Fig. 8:**
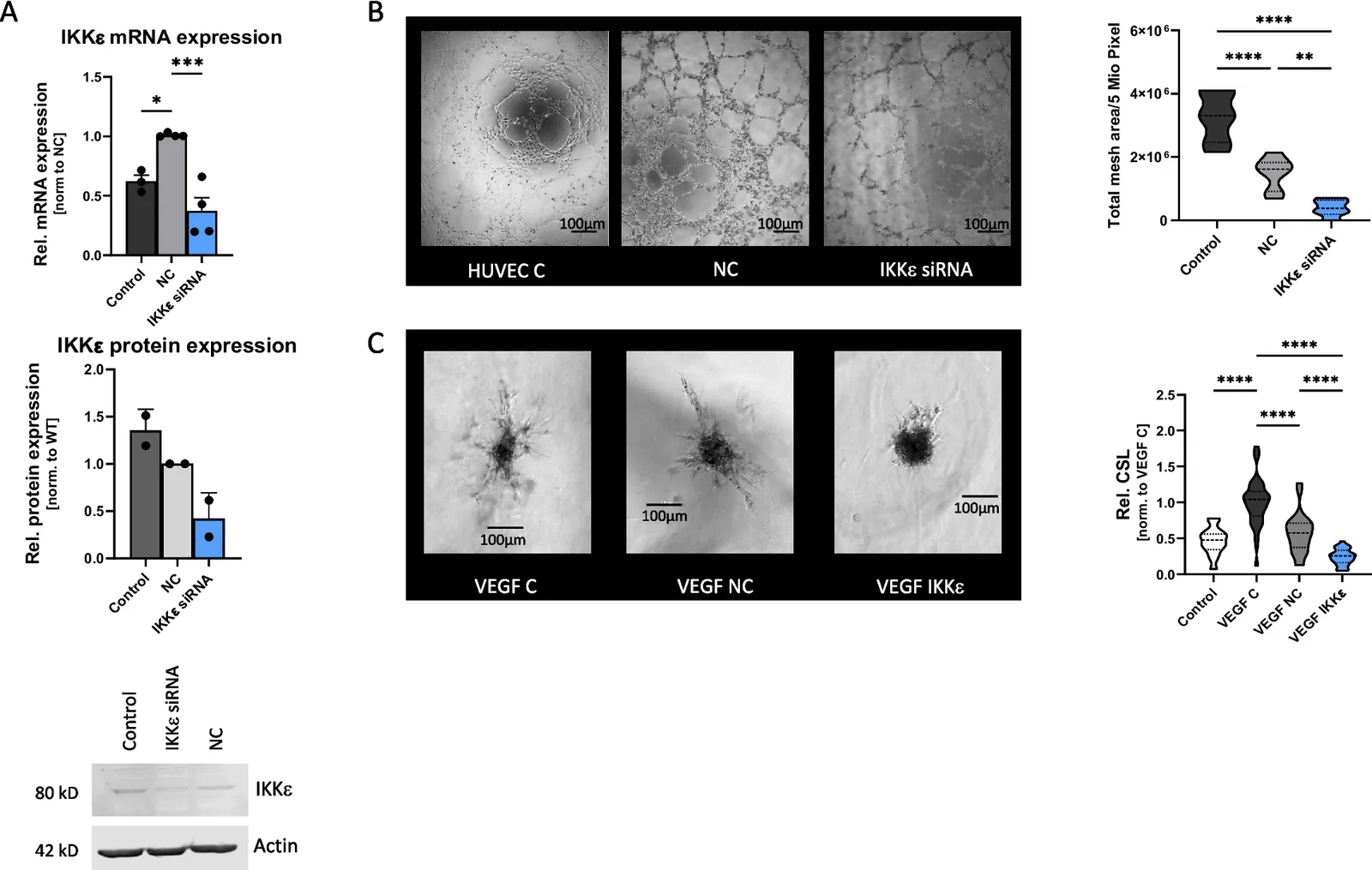
Angiogenesis assay in HUVEC with and without depletion of IKKε by siRNA. **A** IKKε mRNA and protein expression in HUVEC cells after treatment with IKKε siRNA or a scrambled negative control as determined by PCR and Western Blot analysis, PCR: *n* = 3–4/group, Western Blot (*n* = 2/group) (**B**) Tube formation assay, left side: microscopic pictures, right side: analysis of the total mesh area of naïve HUVEC, or HUVEC treated with NC and IKKε siRNA. *n* = 6–7/group. The scale bars represent 100 µm. **C** Spheroid sprout assay, left side: microscopic pictures, right side: analysis of cumulative sprout length (CSL) in spheroids of naïve HUVEC (*n* = 15 spheroids), HUVEC treated with VEGF (*n* = 38 spheroids) HUVEC-NC (*n* = 25 spheroids) and HUVEC-IKKε siRNA (*n* = 38 spheroids). Data were generated in 5 independent biological experiments. The scale bars represent 100 µm. One Way ANOVA with Tukey´s multiple comparisons test, *P < 0.05, **P < 0.01, ***P < 0.001, ****P < 0.0001

To determine the role of IKKε in angiogenesis, we performed tube-formation and spheroid assays using human umbilical vein endothelial cells (HUVEC). These were transfected with IKKε-specific siRNA, while control cells received scrambled RNA (NC). Efficient knock-down of IKKε was confirmed by RT-qPCR and Western Blot analysis. Interestingly, treatment with NC resulted in an increase in IKKε mRNA but not protein, which may reflect off-target effects of the oligonucleotide that influence transcript levels without translating into corresponding changes at the protein level (Fig. 8A). In the tube-formation assay, naïve HUVEC developed an extensive and well-organized vascular network. This network was already impaired in NC-transfected cells, most likely due to the treatment with the transfection reagent. This impairment was significantly more pronounced in IKKε-depleted cells, which developed an incomplete and partially disrupted meshwork. Quantification of the total mesh area revealed a significant reduction in network formation in IKKε-knock-down cells compared with NC controls (Fig. 8B). The spheroid assay yielded comparable results. VEGF stimulation markedly increased sprout formation in untreated control spheroids, as reflected by the cumulative sprout length (CSL). This VEGF-induced response was attenuated by NC treatment and further diminished following IKKε depletion, indicating a robust requirement for IKKε in endothelial sprouting (Fig. 8C).

## Discussion

The protein kinase IKKε has been associated with initiation and progression of melanoma. The majority of studies to date have concentrated on IKKε-mediated mechanisms in tumor cells and showed that inhibition of IKKε is associated with decreased tumor growth. The tumor suppressing mechanisms include inhibition of NF-κB, MAPK and Akt1 pathways, as well as inhibition of autophagy (Möller et al., 2020; Moser et al. 2016). However, previous data also suggested that IKKε is abundantly expressed in T-cells and plays a role in their activation (Peters et al. 2000). Zhang et al. stated that IKKε is a NFATc1 kinase that functions by inhibiting NFATc1 activation and T-cell immune responses. The depletion of IKKε has been demonstrated to increase T-cell activity in vitro and augment T-cell immunity in mice. These effects were associated with a reduction of viral infections and the development of metastatic melanoma in the lungs. It has been hypothesized that these effects are primarily mediated by CD8⁺ T-cells (Zhang et al. 2016). The present study aimed to investigate the impact of IKKε in the melanoma tumor microenvironment, with a particular focus on T-cell regulations and angiogenesis. Our results showed a significant decrease in subcutaneous tumor development in IKKε KO mice. This anti-tumor response was associated with an increase in the number of CD8⁺ cytotoxic and γδ-T-cells while the number of neutrophils decreased. These data suggest a potential correlation between the inhibition of IKKε and the suppression of anti-tumor immunity since augmented levels of cytotoxic lymphocytes such as CD8⁺ T-cells, NK cells and γδ-T-cells, in tumors have been associated with a favorable prognosis in cancer. Conversely, polymorphonuclear cells are frequently discussed as adverse cancer prognostic populations (Gentles et al. 2015; Vakkila and Lotze 2004; Pekarek et al. 1995).

CD8⁺ T-cells are central mediators of anti-tumor immunity, eliminating malignant cells through cytotoxic granules and cytokines such as IFN-γ and tumor necrosis factor α (TNF-α). Although their activation typically depends on major histocompatibility complex (MHC)-I presentation, recent work shows that T-cell-based immunotherapies can remain effective even against MHC-I–deficient melanoma lines (Lerner et al. 2023). γδ-T-cells recognize antigens independently of MHC and are therefore well suited to target tumors with MHC downregulation. Functionally, γδ-T-cells comprise interleukin-(IL-)17–producing subsets that can promote tumor growth and IFN-γ–producing subsets with cytotoxic, anti-tumor activity (Long et al. 2017; Silva-Santos et al. 2019). Furthermore, they can enhance CD8⁺ T-cell responses (Brandes et al. 2005). In melanoma, γδ-T-cell infiltration is increased in human metastatic lesions (Cordova et al. 2012; Campillo et al. 2007) and γδ-T-cell–deficient mice develop tumors more readily, associated with reduced IFN-γ levels (Gao et al. 2003). In our experiments, CD8⁺ and γδ T-cells from wild-type and IKKε-knockout mice showed comparable migration toward B16BL6-conditioned medium and no differences in B16BL6 proliferation, which may reflect the absence of additional cofactors present *in vivo*. However, IKKε-deficient CD8⁺ T-cells released more granzyme B and IFN-γ than wild-type cells, whereas γδ-T-cells showed no such difference. Nevertheless, it is possible that the elevated γδ-T-cell numbers observed in IKKε KO tumors may enhance CD8⁺ T-cell activation *in vivo*. Overall, our findings identify CD8⁺ T-cells as key mediators of tumor suppression following IKKε inhibition, a conclusion supported by in vivo CD8⁺ T-cell depletion, which inhibited the anti-tumor effect in IKKε KO mice. Proteomic profiling of mono- and co-cultured B16BL6 cells revealed expected T-cell-driven regulation of cell-cycle proteins in both genotypes. Notably, CD8⁺ T-cells from IKKε-deficient mice induced a stronger inflammatory response in tumor cells, consistent with their heightened activation and increased expression of inflammatory mediators. OLINK proteome data from ex vivo tumors also revealed significant regulations of multiple proteins including Ccl2 (monocyte chemoattractant protein 1, MCP-1), Cxcl1 (growth-related oncogene, GROa) and Cyr61 which were all decreased in tumors from IKKε KO mice. Ccl2 attracts invasion of M2 macrophages and regulatory T-cells into the tumor leading to suppression of the immune response (Fridlender et al. 2010; Conti and Rollins 2004). In contrast, Ccl2 impedes the infiltration of CD8⁺ T-cells into tumors (Peng et al. 1997). Inhibition of Ccl2 has been associated with the inhibition of melanoma growth in mice (Koga et al. 2008) and augmented cancer immunotherapy in general (Fridlender et al. 2010). The reduced level of Ccl2 in the tumors of IKKε KO mice may therefore also contribute to a better impact of CD8⁺ T-cells. However, a direct functional interaction between Ccl2 and CD8⁺ T-cell activity would require additional experimental validation, for example through an exogenous Ccl2 rescue approach. In addition to their immune-modulatory roles, Ccl2, Cxcl1, and Cyr61 are well-established promoters of angiogenesis (Conti and Rollins 2004; Korbecki et al. 2025; Babic et al. 1998). This raised the hypothesis that IKKε suppression may impair angiogenesis, thereby contributing to reduced tumor growth. Indeed, previous work in a mouse glioblastoma model suggests that IKKε depletion diminishes angiogenesis by lowering VEGF signaling via the Akt/FOXO3A pathway (Zhu et al. 2023). ELR⁺ chemokines such as Cxcl1–3 positively regulate angiogenesis through Cxcr2 on endothelial cells (Addison et al. 2000) and Cxcl1, upregulated by NF-κB hyperactivation in melanoma, supports tumor progression (Strieter et al. 1995; Haghnegahdar et al. 2000; Wood and Richmond 1995). Beyond angiogenesis, Cxcl1 promotes tumor proliferation, migration, and neutrophil recruitment via Cxcr2 (Dhawan and Richmond 2002; Moser et al. 1990; Korbecki et al. 2022). Thus, the reduced levels of Cxcl1 and Cxcl2 in IKKε-deficient tumors likely contribute to both lower neutrophil infiltration and reduced angiogenesis, the latter supported by decreased CD31^+^ endothelial cells. At early tumor stages, when tumor sizes were comparable, IKKε-deficient tumors also showed reduced expression of key angiogenic regulators including HIF-1α, VEGF, and MMP-9. HIF-1α, induced by tumor hypoxia, drives VEGF expression and angiogenesis (Blagosklonny 2004; Rey et al. 2017), while MMP-9 facilitates extracellular matrix degradation, invasion, and vascular remodeling (Yu and Stamenkovic 2000). These changes are consistent with reduced NF-κB activity in IKKε KO mice, and endothelial cell culture experiments further support a role for IKKε in regulating angiogenesis. At later tumor stages, HIF-1α was no longer reduced and VEGF was upregulated, indicating a compensatory re-activation of VEGF-mediated signaling. These findings suggest a temporal shift in the angiogenic program, with IKKε knockout primarily impairing early angiogenesis. The absence of a corresponding increase in HIF1α implies that the late VEGF rise may occur at least partially through HIF-1α-independent pathways. However, although VEGF increased, tumor growth was not restored, suggesting that the early angiogenic defect creates a lasting growth disadvantage. Moreover, the sustained reduction of Cxcl1, CD31, and MMP-9 supports that this VEGF upregulation does not lead to a mature vascular network but instead reflects an incomplete or qualitatively altered angiogenic response (Liu et al. 2025; Tamura et al. 2020; Yang et al. 2024; De Palma et al. 2017).

Despite the promising results, our study has several limitations. Even though we identified regulatory changes consistent with reduced angiogenesis, decreased neutrophil infiltration, and enhanced T-cell-mediated antitumor activity, some of these findings remain correlative. Rescue approaches, such as restoring Cxcl1, VEGF, or IKKε, would help clarifying the specific contribution of IKKε to melanoma progression. Likewise, conditional IKKε knockout in defined cell populations, such as endothelial cells or distinct T-cell subsets, would allow a more precise identification of IKKε-dependent target cells. These experiments were not feasible here, as suitable IKKε-Cre mouse lines are currently unavailable.

## Conclusion

In summary, our recent (Möller et al., 2020; Moser et al. 2016) and current data indicate that IKKε in tumor cells and in the tumor microenvironment is involved in the initiation and progression of melanoma. Its inhibition decreases melanoma tumor growth by impeding NF-κB and MAPK pathways in the tumor cells and by augmenting the presence of cytotoxic CD8⁺ T-cells. Furthermore, blocking IKKε decreases angiogenic markers, which might also contribute to the anti-tumor effects observed. In conclusion, IKKε could be a promising target for melanoma treatment, particularly in combinatorial approaches. The hypothesis is further substantiated by studies demonstrating the efficacy of IKKε/TBK1 inhibitors as therapeutic agents for the treatment of melanomas harboring mutations in NRAS and BRAF (Eskiocak et al. 2017). Nevertheless, future clinical studies will be required to clarify whether IKKε could serve as a biomarker or therapeutic target in human melanoma.

## Supplementary Information

Supplementary Material 1.

## Data Availability

All data supporting the findings of this study are available within the paper and its Supplementary Information.

## Abbreviations

BRAF: B-rapidly accelerated fibrosarcoma
CBA: Cytometric bead array
CCL: CC-chemokine-ligand
CCR: C-C-chemokine-receptor
CSL: Cumulative sprout length
CXCL: C-X-C motiv chemokine ligand
CXCR: C-X-C-motiv-chemokine receptor
Cyr61: Cysteine-rich angiogenic inducer 61
FACS: Fluorescence-activated cell sorting
GAPDH: Glyceraldehyde 3-phosphate dehydrogenase
GROa: Growth-regulated oncogene alpha
HIF-1a: Hypoxia-inducible factor- 1 alpha
HUVEC: Human umbilical vein endothelial cells
IL: Interleukin
IFN-γ: Interferon- γ
IKKε: Inhibitor of nuclear factor kappa-B kinase epsilon
MAPK: Mitogen-activated protein kinase
MHC: Major histocompatibility complex
MCP-1: Monocyte chemoattractant protein 1
MMP: Matrix-metalloproteinase
NC: Negative control
NFAT: Nuclear factor of activated T-cells
NRAS: Neuroblastoma RAS
TBK1: TANK-binding kinase 1
TME: Tumor microenvironment
TNF α: Tumor necrosis factor α
VEGF: Vascular endothelial growth factor
WT: Wildtype
