# Supplementary material for: The protein kinase IKK epsilon shapes the melanoma tumor microenvironment by modulating angiogenesis and the T-cell immunity
Source: Mol Med. 2026 Sep 30;32:167. doi: 10.1186/s10020-026-01651-w (PMC13625450; doi:10.1186/s10020-026-01651-w)
Supplement: Supplementary file 1 [file 10020_2026_1651_MOESM1_ESM.docx]

**Supplementary material**

***Suppl. Figure 1:*** *Gating Strategy and single channel photographs of human melanoma*

(A) Gating strategy for phenoptics staining, (B) Overview of all antibody staining on a representative slice, merge and single channels photographs, Scale Bar: 20 µm, (C) Colocalization of CD3 and IKKε, merge and single channel photographs, Scale Bar: 20 µm, (D) Colocalization of S100 and IKKε, merge and single channel photographs, Scale Bar: 20 µm.

***Suppl. Figure 2:*** *Immune cell levels in melanoma of WT and IKKε knock-out mice*

*(A) Gating Strategy for FACS analysis, (B)* Immune cell numbers of CD4^+^ T-cells, regulatory T-cells (T_reg_), macrophages, dendritic cells and Natural Killer (NK) cells in the tumors as determined by flow cytometry. Cell counts were normalized to the number of living immune cells. n = 6/group; Student´s t-test, *(B)* Immune cell numbers with cell counts were normalized to the tumor mass [g], n = 6/group; Student´s t-test.

***Suppl. Figure 3:*** *Mathematical extrapolation of the tumors in the CD8a depletion experiment*

CD8⁺ T-cells were depleted in wildtype and IKKε KO mice by intraperitoneal injection of anti-mouse CD8α antibody. IgG injection served as isotype control. Left panel: Tumor growth over a period of 21 days, Two-way ANOVA with Tukey´multiple comparisons test. Right panel: calculated tumor mass at the end of the experiment. One-way ANOVA with Tukey post hoc analysis. WT + Isotype ctrl: n = 6 mice, WT + anti-CD8-TCR: n = 6 mice, IKKε KO + Isotype ctrl: n = 5 mice. IKKε KO + anti-mouse CD8α: n = 6 mice.

***Suppl. Figure 4:*** *Proteome analysis of B16 cell with and without CD8 T-cell coculture*

B16BL6 were either cultured as monoculture or as co-culture with CD8⁺ T-cells from wildtype and IKKε KO mice. (A) Multiple Scatter Blot showing correlations between replicates and samples, respectively. (B) Histograms showing normal distribution of the respective samples. (C) Volcano plot showing no significant differences between co-culture of B16BL6 cells with either wildtype or IKKε KO T-cells. n = 3 for B16BL6-T-cell co-cultures

**Suppl. Table 1:** Antibodies used for FACS analysis of migrated cells and spleen

| **Target** | **Fluorophore** | **Distributor** | **Cat.-No.** |
| --- | --- | --- | --- |
| CD3 | PE-CF594 | BD Biosciences | 562286 |
| CD4 | BV711 | BD Biosciences | 563050 |
| CD8 | BV650 | BioLegend® | 100742 |
| CD45 | AF700 | BioLegend® | 103128 |
| γδ-TCR | PerCP/Cy5.5 / BB700 | BioLegend® | 118118 |
| TCR Vγ3 | PE | BioLegend® | 137504 |
| TCR Vγ1.1 | FITC / BB515 | BioLegend® | 141104 |
| TCR Vγ2 | APC | BioLegend® | 137708 |
| TCR Vδ4 | PE-Vio770/ PE-Cy7 | Miltenyi Biotec | 130-105-677 |

**Suppl. Table 2:** Antibodies used for FACS analysis of melanoma

| **Target** | **Fluorophore** | **Distributor** | **Cat.-No.** |
| --- | --- | --- | --- |
| CD3 | PE-CF594 | BD Biosciences | 562286 |
| CD4 | BV711 | BD Biosciences | 563050 |
| CD8 | BV650 | BioLegend® | 100742 |
| CD11b | BV605 | BioLegend® | 101257 |
| CD11c | BV711 | BD Biosciences | 563048 |
| CD19 | APC-H7 (APC-Cy7) | BioLegend® | 115558 |
| CD31 | PE-Cy7 | Invitrogen/Thermo Fisher Scientific | 25-0311-82 |
| CD45 | VioBlue (BV421) | Miltenyi Biotec | 130-118-953 |
| CD90.2 | PE | Miltenyi Biotec | 130-102-489 |
| CD117 | APC-eFluor780 | Invitrogen (Thermo Fisher Scientific) | 47-1172-82 |
| CD146 | AlexaFluor488 | BioLegend® | 134708 |
| CD324 | AlexaFluor 647 | BioLegend® | 147308 |
| CD326 | BV711 | BD Biosciences | 563134 |
| GITR | FITC | BioLegend® | 126308 |
| SiglecH | FITC | BioLegend® | 129604 |
| F4/80 | PE-Cy7 | BioLegend® | 123114 |
| γδ-TCR | APC | BioLegend® | 118116 |
| HLA-DR (MHC II) | APC | BioLegend® | 107614 |
| Ly-6C | PerCP-Cy5.5 (BB700A) | BioLegend® | 128012 |
| Ly-6G | APC-Cy7 | BioLegend® | 127624 |
| NK1.1 | BV510 (BV421) | BD Biosciences | 563096 |

**Suppl. Table 3:** Olink target 96 inflammation

| **UniProt ID** | **Gene** | **Protein name** | |
| --- | --- | --- | --- |
| P30203 | CD6 | T-cell differentiation antigen CD6 | |
| P21583 | KITLG | Kit ligand |  |
| Q14116 | IL18 | Interleukin-18 | |
| Q13291 | SLAMF1 | Signaling lymphocytic activation molecule | |
| P01135 | TGFA | Protransforming growth factor alpha | |
| Q99616 | CCL13 | C-C motif chemokine 13 | |
| P51671 | CCL11 | Eotaxin |  |
| O43557 | TNFSF14 | Tumor necrosis factor ligand superfamily member 14 | |
| Q9GZV9 | FGF23 | Fibroblast growth factor 23 | |
| Q13651 | IL10RA | Interleukin-10 receptor subunit alpha | |
| P13236 | CCL4 | C-C motif chemokine 4 | |
| P12034 | FGF5 | Fibroblast growth factor 5 | |
| P42702 | LIFR | Leukemia inhibitory factor receptor | |
| Q9NSA1 | FGF21 | Fibroblast growth factor 21 | |
| Q99731 | CCL19 | C-C motif chemokine 19 | |
| Q13261 | IL15RA | Interleukin-15 receptor subunit alpha | |
| Q08334 | IL10RB | Interleukin-10 receptor subunit beta | |
| Q8N6P7 | IL22RA1 | Interleukin-22 receptor subunit alpha-1 | |
| Q13478 | IL18R1 | Interleukin-18 receptor 1 | |
| Q9NZQ7 | CD274 | Programmed cell death 1 ligand 1 | |
| P01138 | NGF | Beta-nerve growth factor | |
| P42830 | CXCL5 | C-X-C motif chemokine 5 | |
| P03956 | MMP1 | Interstitial collagenase | |
| O14788 | TNFSF11 | Tumor necrosis factor ligand superfamily member 11 | |
| Q969D9 | TSLP | Thymic stromal lymphopoietin | |
| P60568 | IL2 | Interleukin-2 | |
| P15692 | VEGFA | Vascular endothelial growth factor A | |
| P80098 | CCL7 | C-C motif chemokine 7 | |
| P39905 | GDNF | Glial cell line-derived neurotrophic factor | |
| Q9H5V8 | CDCP1 | CUB domain-containing protein 1 | |
| Q9BZW8 | CD244 | Natural killer cell receptor 2B4 | |
| P13232 | IL7 | Interleukin-7 | |
| O00300 | TNFRSF11B | Tumor necrosis factor receptor superfamily member 11B | |
| P01137 | TGFB1 | Transforming growth factor beta-1 proprotein | |
| P00749 | PLAU | Urokinase-type plasminogen activator | |
| P05231 | IL6 | Interleukin-6 | |
| P09341 | CXCL1 | Growth-regulated alpha protein | |
| Q9P0M4 | IL17C | Interleukin-17C | |
| Q16552 | IL17A | Interleukin-17A | |
| O14625 | CXCL11 | C-X-C motif chemokine 11 | |
| O15169 | AXIN1 | Axin-1 |  |
| P50591 | TNFSF10 | Tumor necrosis factor ligand superfamily member 10 | |
| Q9UHF4 | IL20RA | Interleukin-20 receptor subunit alpha | |
| Q07325 | CXCL9 | C-X-C motif chemokine 9 | |
| P28325 | CST5 | Cystatin-D |  |
| P14784 | IL2RB | Interleukin-2 receptor subunit beta | |
| P01583 | IL1A | Interleukin-1 alpha | |
| P13725 | OSM | Oncostatin-M | |
| P13500 | CCL2 | C-C motif chemokine 2 | |
| P10145 | CXCL8 | Interleukin-8 | |
| Q8NFT8 | DNER | Delta and Notch-like epidermal growth factor-related receptor | |
| Q9NRJ3 | CCL28 | C-C motif chemokine 28 | |
| Q8IXJ6 | SIRT2 | NAD-dependent protein deacetylase sirtuin-2 | |
| Q9NYY1 | IL20 | Interleukin-20 | |
| Q13541 | EIF4EBP1 | Eukaryotic translation initiation factor 4E-binding protein 1 | |
| P02778 | CXCL10 | C-X-C motif chemokine 10 | |
| P80162 | CXCL6 | C-X-C motif chemokine 6 | |
| P49771 | FLT3LG | Fms-related tyrosine kinase 3 ligand | |
| P80511 | S100A12 | Protein S100-A12 | |
| P10147 | CCL3 | C-C motif chemokine 3 | |
| P55773 | CCL23 | C-C motif chemokine 23 | |
| P01375 | TNF | Tumor necrosis factor | |
| P22301 | IL10 | Interleukin-10 | |
| P09238 | MMP10 | Stromelysin-2 | |
| Q5T4W7 | ARTN | Artemin |  |
| P35225 | IL13 | Interleukin-13 | |
| Q13007 | IL24 | Interleukin-24 | |
| P29460 | IL12B | Interleukin-12 subunit beta | |
| P06127 | CD5 | T-cell surface glycoprotein CD5 | |
| P14210 | HGF | Hepatocyte growth factor | |
| P25942 | CD40 | Tumor necrosis factor receptor superfamily member 5 | |
| P01579 | IFNG | Interferon gamma | |
| P09603 | CSF1 | Macrophage colony-stimulating factor 1 | |
| P01374 | LTA | Lymphotoxin-alpha | |
| P00813 | ADA | Adenosine deaminase | |
| P05113 | IL5 | Interleukin-5 | |
| O95630 | STAMBP | STAM-binding protein | |
| P50225 | SULT1A1 | Sulfotransferase 1A1 | |
| P78556 | CCL20 | C-C motif chemokine 20 | |
| O43508 | TNFSF12 | Tumor necrosis factor ligand superfamily member 12 | |
| O95760 | IL33 | Interleukin-33 | |
| P20783 | NTF3 | Neurotrophin-3 | |
| P78423 | CX3CL1 | Fractalkine |  |
| O15444 | CCL25 | C-C motif chemokine 25 | |
| Q14790 | CASP8 | Caspase-8 |  |
| P80075 | CCL8 | C-C motif chemokine 8 | |
| Q99748 | NRTN | Neurturin |  |
| P15018 | LIF | Leukemia inhibitory factor | |
| P05112 | IL4 | Interleukin-4 | |
| O95750 | FGF19 | Fibroblast growth factor 19 | |
| Q07011 | TNFRSF9 | Tumor necrosis factor receptor superfamily member 9 | |
| P01732 | CD8A | T-cell surface glycoprotein CD8 alpha chain | |

**Suppl. Table 4:** Multivariable regression table for human melanoma

| **Variable** | **Hazard Ratio** | **CI lower** | **CI upper** | **P-value** |
| --- | --- | --- | --- | --- |
| Expression high | 2.73 | 1.16 | 6.43 | 0.0212 |
| Age | 1.00 | 0.971 | 1.03 | 1.0 |
| Stage group III-IV | 2.54 | 1.13 | 5.71 | 0.0242 |
